# Decoding the Primacy of Transportation Emissions of Formaldehyde Pollution in an Urban Atmosphere

**DOI:** 10.3390/toxics13080643

**Published:** 2025-07-30

**Authors:** Shi-Qi Liu, Hao-Nan Ma, Meng-Xue Tang, Yu-Ming Shao, Ting-Ting Yao, Ling-Yan He, Xiao-Feng Huang

**Affiliations:** 1 Key Laboratory for Urban Habitat Environmental Science and Technology, Peking University Shenzhen Graduate School, Shenzhen 518055, China; liushiqi67@stu.pku.edu.cn (S.-Q.L.); mahaonan@stu.pku.edu.cn (H.-N.M.);; 2 Shenzhen Academy of Metrology and Quality Inspection, Shenzhen 518107, China

**Keywords:** HCHO, PMF, machine learning, transportation emission, photochemical pollution

## Abstract

Understanding the differential impacts of emission sources of volatile organic compounds (VOCs) on formaldehyde (HCHO) levels is pivotal to effectively mitigating key photochemical radical precursors, thereby enhancing the regulation of atmospheric oxidation capacity (AOC) and ozone formation. This investigation systematically selected and analyzed year-long VOC measurements across three urban zones in Shenzhen, China. Photochemical age correction methods were implemented to develop the initial concentrations of VOCs before source apportionment; then Positive Matrix Factorization (PMF) modeling resolved six primary sources: solvent usage (28.6–47.9%), vehicle exhaust (24.2–31.2%), biogenic emission (13.8–18.1%), natural gas (8.5–16.3%), gasoline evaporation (3.2–8.9%), and biomass burning (0.3–2.4%). A machine learning (ML) framework incorporating Shapley Additive Explanations (SHAP) was subsequently applied to evaluate the influence of six emission sources on HCHO concentrations while accounting for reaction time adjustments. This machine learning-driven nonlinear analysis demonstrated that vehicle exhaust nearly always emerged as the primary anthropogenic contributor in diverse functional zones and different seasons, with gasoline evaporation as another key contributor, while the traditional reactivity metric method, ozone formation potential (OFP), tended to underestimate the role of the two sources. This study highlights the primacy of strengthening emission reduction of transportation sectors to mitigate HCHO pollution in megacities.

## 1. Introduction

Formaldehyde (HCHO) is among the most abundant reactive carbonyls in the troposphere and serves as a key precursor of HO_x_ radicals, thereby enhancing the atmospheric oxidative capacity (AOC) and driving tropospheric ozone (O_3_) production [1,2,3]. Primary HCHO emissions arise from anthropogenic activities, including vehicular and industrial sources, and from natural sources such as biogenic emissions and biomass burning [4,5]. Furthermore, the involvement of volatile organic compounds (VOCs) in the photochemical oxidation processes mediated by OH/NO_3_/O_3_ in the atmosphere constitutes an indispensable secondary mechanism for HCHO formation [6,7]. The photolytic decomposition of HCHO predominantly yields OH· radicals, while its oxidation indirectly fosters HO_2_ radical production through NO-to-NO_2_ conversion, both processes critically strengthening the AOC [8,9]. Given the severe ozone pollution in the Pearl River Delta (PRD) and the critical role of HCHO as both a significant photochemical byproduct and an active participant in radical-driven processes, it is imperative to investigate HCHO in this region [10,11,12].

Moreover, HCHO has been designated as a Group I human carcinogen by the International Agency for Research on Cancer (IARC) [13] and is widely recognized for its well-documented carcinogenicity and mutagenicity [7], posing a substantial threat to public health. Relevant studies have demonstrated that outdoor sources of HCHO (e.g., motor vehicle emissions and photochemical reactions) constitute a pivotal factor in shaping indoor HCHO pollution and its consequent health hazards [14,15]. Hence, establishing effective evidence-based strategies for HCHO control is essential to improving atmospheric photochemical conditions and enhancing public health in both indoor and outdoor settings.

The commonly used methods for source apportionment of HCHO include Multiple Linear Regression (MLR) [16], Photochemical Age-Based Parameterization (PCAP) method [3,10,11,12], and the Positive Matrix Factorization (PMF) model [17]. Cui et al. [18] pioneered a concurrent application of MLR, PCAP, and PMF models for HCHO source apportionment, revealing method-specific discrepancies in secondary contributions while highlighting the inherent limitations of individual approaches. Notably, the complex nonlinear relationships between HCHO and its emission sources challenge traditional source apportionment methods. It warrants further emphasis that VOC sources may have undergone photochemical consumption in the atmosphere prior to observation, rendering them unrepresentative of initial states [19,20]. Neglecting uncorrected photochemical losses systematically biases source apportionment and can exacerbate discrepancies between inferred emission sources and actual emissions [21,22,23].

Currently, advancements in AI have transcended traditional computational algorithms, enabling computers to progressively develop autonomous learning capabilities, which were the quintessential human traits [24]. Trained AI models have achieved subseasonal forecasting accuracy of complex events, such as global weather prediction, which was regarded as a significant breakthrough in the scientific community [25,26]. Machine learning (ML), as a transformative subfield of AI methodology, is increasingly providing novel analytical frameworks in environmental sciences for predicting and simulating pollutant behaviors [24,27,28]. ML has unique advantages through adaptive weight optimization in addressing complex nonlinear problems and identifying latent variables in emission matrices, demonstrating robust performance in terms of temporal and cost efficiency [29,30]. Moreover, with the advent of the Shapley Additive Explanations (SHAP) algorithm, the model interpretability of typically “black-box” ML has been substantially enhanced. The SHAP algorithm quantifies feature contributions of each factor by elucidating the interactions and dependencies among driving variables within ML models according to coalitional game theory [31]. The integrated application of these technologies holds the potential to elucidate the nonlinear dynamic transformations of HCHO in atmospheric photochemical pollution.

This study introduced an innovative PMF-ML framework to assess the impact of primary VOC emissions on HCHO concentrations. In contrast to conventional approaches that typically perform direct source apportionment of HCHO, this study first independently conducted PMF-based source apportionment of photochemically adjusted VOCs representing primary emissions. Subsequently, machine learning techniques were employed to establish a nonlinear correlation between VOCs and HCHO. This novel methodology offered a new perspective for evaluating the influence of emissions on HCHO levels, thereby providing valuable insights to inform the development of targeted photochemical pollution control strategies.

## 2. Materials and Methods

### 2.1. Study Areas and Photochemical Age Correction

**Figure 1:**
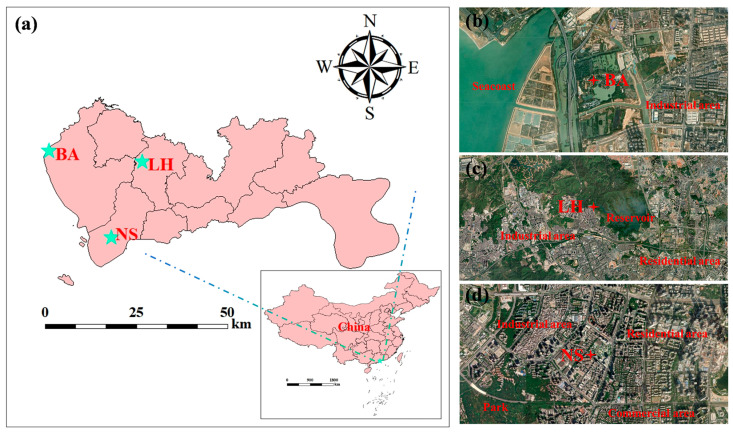
(**a**) The location conditions of the study areas in the Shenzhen region of China. Satellite maps of monitoring sites from Google Earth in (**b**) Bao’an district (BA), (**c**) Longhua district (LH), and (**d**) Nanshan district (NS).

Three monitoring sites were meticulously selected to epitomize quintessential industrial and urban typologies characterized by intricate traffic networks and substantial population densities. These archetypal stations conducted synchronized and real-time measurements of 116 VOC species and meteorological parameters in Shenzhen (Figure 1). Comprehensive geospatial characteristics, including coordinates and contextual information for each site, are presented in Table S1. The observational period spanned from 1 July 2023 to 30 June 2024, which ensured a full annual cycle of data collection. In addition, the comprehensive descriptions of analytical instruments and the principles of quality control are listed in Texts S1 and S2, respectively. Meanwhile, the daytime period (07:00–18:00) was captured when photolytic effects were pronounced by the atmospheric photolysis rate analyzer (PFS-100, Hangzhou Juguang, China) across the three Shenzhen sites, and this period was selected for photochemical age corrections of all VOC species included in the analysis of the source apportionment (detailed methods are illustrated in Text S3 and Figure S1) [20,21,32,33,34,35].

### 2.2. Positive Matrix Factorization Model

Source apportionment and component analysis of VOC species were conducted utilizing the Positive Matrix Factorization (PMF v5.0) model [36,37]. PMF utilizes the concentrations of representative species combined with associated uncertainties (Unc) as model inputs. Species selection followed three stringent criteria: (1) prolonged photochemical lifetime, (2) source-specific tracer characteristics, and (3) high detection frequency [38]. Ultimately, 25 species were identified (Tables S2 and S3), and concentrations were normalized using photochemical age correction. The factor evaluations and model fitting assessments were meticulously executed in accordance with the PMF v5.0 User Guide (US EPA, 2014), and comprehensive methodologies and results are detailed in Text S4 [38,39,40]. Furthermore, the ozone formation potential (OFP) metric [41,42] was applied to evaluate the differential contributions of VOCs with varying reactivities to O_3_ formation, thereby quantifying the contributions of both VOC species and PMF-resolved sources to photochemical processes. Further methodological details are provided in Texts S5 and S6.

### 2.3. Machine Learning Model

This study developed machine learning models using Python 3.11 (Anaconda v2.1.1) with key packages including scikit-learn (v1.2.2) and XGBoost (v1.7.3). The Extreme Gradient Boosting (XGBoost) regression model [28,29] was developed to predict ambient HCHO concentrations using six PMF-derived source contribution variables and four meteorological parameters (temperature [T], relative humidity [RH], photolysis rate [JV], and wind speed [WS]) as independent predictors (number of decision trees = 300–1000, learning rate = 0.001–0.03, max depth = 3–10). The PMF results were based on hourly VOC data apportioned by principal sources, with photochemical aging taken into account. For robustness validation, Light Gradient Boosting Machine (LightGBM) and Random Forest (RF) models were established with identical predictor–response configurations [30]. Following the removal of incomplete data records (<10% missing values), the dataset was stratified into training (80%) and testing (20%) sets, with performance evaluation through R^2^, root mean square error (RMSE), mean square error (MSE), and mean absolute error (MAE) metrics. Hyperparameter optimization was conducted through Bayesian optimization using the BayesSearchCV algorithm from the scikit-optimize package (v0.10.1), implementing 50 iterations with five-fold cross-validation [43]. This approach replaced conventional grid search for more efficient hyperparameter space exploration. The framework and mathematical principles of machine learning and Bayesian optimization are comprehensively documented in Text S7. All above models exhibited robust predictive performance (testing set R^2^: 0.79–0.83; RMSE: 0.79–0.92), with XGBoost consistently achieving the superior performance bounds (R^2^: 0.80–0.83; RMSE: 0.79–0.85). To assess the relative contributions of predictors to the dependent variable HCHO, feature importance was analyzed using SHAP values computed with the Python SHAP package (v0.42.1) [31]. All model-related Python packages are permanently archived on the Python Package Index (PyPI) at https://pypi.org, accessed on 1 March 2025, ensuring computational reproducibility.

## 3. Results

### 3.1. Overview of VOC Components and Activities

**Figure 2:**
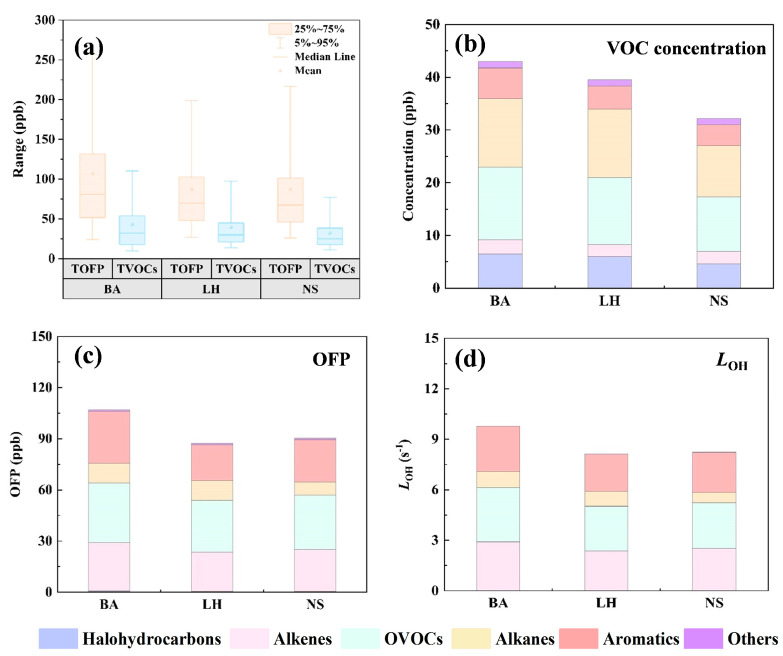
(**a**) Box plots of TVOCs and TOFP. The compositions of (**b**) concentration, (**c**) OFP, and (**d**) LOH of VOC species.

The concentration levels of atmospheric pollutants are predominantly governed by the intensity of emission sources, prevailing meteorological conditions, and photochemical reaction dynamics [44]. Herein, the annual mean concentrations of total measured VOCs (TVOCs) at three representative Shenzhen sites were measured as 43.0 ± 45.0 ppb (BA), 39.6 ± 33.8 ppb (LH), and 32.2 ± 23.8 ppb (NS), respectively (Figure 2a). The annual component time series (Figures S2–S4) exhibited a uniform trend across all three sites. Specifically, oxygenated VOCs (OVOCs) predominated in traditional spring and summer compositions, likely attributable to conducive photochemical conditions, including elevated temperatures and increased solar radiation, which facilitated the secondary formation of OVOCs [10,12]. Conversely, alkanes and aromatics exhibited higher proportions during autumn and winter when photochemical activities were subdued, causing high-reactive aromatics to further accumulate, while the presence of low-reactive alkanes indicated heightened vehicular emissions [22]. These findings offered a nuanced understanding of VOC concentration fluctuations throughout Shenzhen. Synchronously, an overview of key meteorological parameters is described in Text S8.

As illustrated in Figure 2b, OVOCs were the dominant VOC species at the BA site, followed by alkanes and halohydrocarbons, with solvent emissions from nearby industrial activities being a major influence [45]. In contrast, at the LH site, alkanes and OVOCs exhibited comparable dominance, reflecting the intricate emissions from vehicular activities and industrial solvents prevalent in the southern and western regions. At the NS site, OVOCs and alkanes again dominated, with acetone (9.20%) and acetaldehyde (7.37%) being the predominant species at NS (Figure S4). Acetaldehyde and acetone are primary OVOCs emitted from diesel and gasoline vehicle exhaust, respectively [3,46], implying that vehicle exhaust significantly shapes the VOC profile at NS [30]. Comprehensive details on VOC concentration levels across the three sites are provided in Table S2.

Annual mean total OFP (TOFP) reached 107.1 ± 103.5 ppb (BA), 87.3 ± 69.7 ppb (LH), and 90.3 ± 76.8 ppb (NS), respectively (Figure 2a). VOC groups such as OVOCs, alkenes, and aromatics consistently dominated the contributions to TOFP at three sites (Figure 2c). The most influential species included acetaldehyde, n-hexanal, ethylene, propylene, isoprene, toluene, and m,p-xylene, exhibiting similar composition patterns at all locations (Figure S5). In terms of OH· reactivity, OVOCs were the predominant contributors, followed by alkenes, aromatics, and alkanes in Figure 2d and Figure S5. At all three sites, the substantial proportions of styrene and isoprene underscored the significant roles of vehicular emissions and biogenic releases in modulating atmospheric oxidative capacity through OH· consumption [11,47].

### 3.2. Analysis Characteristics of VOC Contribution Apportionment

After species fingerprint screening (Table S3), factor diagnostics and model robustness validation (Table S4), the PMF model identified five anthropogenic and one biogenic emission sources after photochemical age correction in the daytime (7:00–18:00) across three monitoring sites. The anthropogenic sources included biomass burning (BB), gasoline evaporation (GE), solvent usage (SU), natural gas (NG), and vehicle exhaust (VE), while the biogenic source (BE) was characterized by isoprene as the representative tracer. Source-specific profiles are depicted in Figure S6.

The first source was distinguished by substantial contributions from chloromethane, acetonitrile, and dichloromethane, which were established tracers of biomass burning [48,49]. Additionally, minor quantities of ethane (secondary combustion indicators) and ethylene that were associated with combustion emissions were detected [46]. Consequently, this source was classified as biomass burning (BB). The second source was predominantly composed of isopentane, n-pentane, and methyl tert-butyl ether (MTBE), where MTBE is considered a common gasoline additive, while isopentane and n-pentane are principal constituents of gasoline [50,51] and supplemented by minor contributions from propane and butane related to petroleum products [45]. Complementarily, the ratio of isopentane/n-pentane at the three sites was 2.45, 2.83, and 2.26 (Table S5), falling within the typical range for gasoline evaporation (1.8–4.0) [22], thus leading to the identification of this source as gasoline evaporation (GE). The third source featured representative aromatic hydrocarbons commonly associated with solvent usage, such as benzene, toluene, ethylbenzene, and m,p-xylene [23,49]. This source was designated as solvent usage (SU), which mainly includes solvent usage from industries, especially in the industrial areas of Shenzhen. The fourth source encompassed ethane, propane, ethylene, propylene, and acetylene, along with minor amounts of isobutane, n-butane, and dichloromethane. Ethylene and propylene are combustion byproducts [46], ethane is a significant emission from natural gas combustion [52,53], and acetylene is closely linked to combustion processes [54]. Meanwhile, the ratio of isobutane/n-butane exceeding 0.6 (Table S5) across all sites indicated the predominance of natural gas combustion [22]. Thus, this source was identified as natural gas (NG), aligning with the clean energy infrastructure of Shenzhen, which primarily utilizes natural gas [45], and this identification is corroborated by similar profiles in other natural gas source studies [22,55]. Further validation of the fourth source was achieved through offline VOC measurements under simulated residential natural gas combustion using a GC-MS system (GCMS 8890-5977B, Agilent, Santa Clara, CA, USA). The VOC source profile obtained from three independent experiments post-background correction (Figure S7) was congruent with the PMF-resolved source profile. The fifth source was dominated by 2-methylpentane, 3-methylpentane, styrene, and various alkanes, where 2-methylpentane and 3-methylpentane are major components of vehicle emissions [56], while C_2_-C_3_ alkanes and alkenes are pivotal exhaust species from gasoline vehicles [3,22]. Long-chain alkanes of C_8_-C_10_ are primarily linked to diesel engine emissions [19], and styrene serves as a tracer for vehicle exhaust [47]. The ratio of propane/ethane below 3.0 across all sites substantiated the influence of vehicle emissions [22]. Therefore, this source was classified as vehicle exhaust (VE).

**Figure 3:**
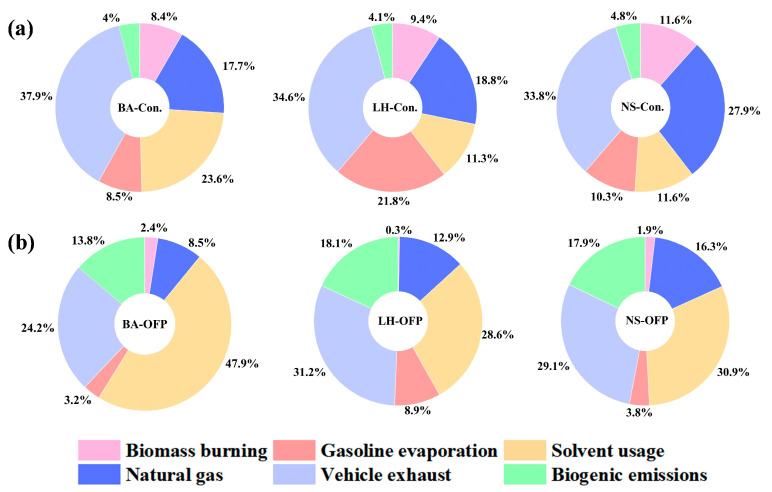
The daytime (7:00–18:00) emission source composition of (**a**) VOCs concentration and (**b**) OFP.

Figure 3a depicts the source contributions to VOC concentrations and OFP at the three sites. The concentrations in source composition of all three sites were dominated by VE, accounting for 37.9% at BA, 34.6% at LH, and 33.8% at NS, respectively. However, the potential photochemical pollution contribution from these sources could not be fully assessed solely based on their concentrations due to the differences in reactivity among emission sources. An examination of OFP contributions across emission sources, as presented in Figure 3b, reveals that SU was the predominant contributor at BA and NS, accounting for 47.9% and 30.9% of TOFP, respectively, while VE was the leading source at LH, contributing 31.2%. Among the three monitoring sites, SU and VE consistently ranked among the most significant contributors. Notably, SU emerged as the dominant anthropogenic source at BA, likely due to the high density of industrial parks and related facilities in the area. The proportions of biogenic emissions were 13.8%, 18.1%, and 17.9% at three sites, although their contribution was proportionally trivial with respect to anthropogenic sources, despite the dominance of BE, which was further accentuated post-photochemical correction [22,57].

Overall, while conventional PMF modeling offered preliminary insights into emission source structures, especially following photochemical age correction, PMF highlighted limitations in resolving the nuanced differences and nonlinear relationships of source contributions to photochemical reactions. Additionally, the potential influence of meteorological factors, regional pollutant transport from other areas, and possible overestimation of actual pre-photochemical correction contributions requires further mechanistic investigation and exploration.

### 3.3. Identification of the Critical Impact of Factors by Machine Learning

Upon being released into the atmosphere, VOCs participate in a cascade of complex photochemical reactions, producing a series of photo-oxidative radicals at successive stages, which eventually culminate in ozone formation. Among the photochemical products, HCHO plays a pivotal intermediary role by amplifying HO_2_ radical formation through its photolysis and subsequent oxidation pathways. Model simulations and RIR-based sensitivity analyses further demonstrate that secondary HCHO, predominantly derived from CH_3_CHO, substantially accelerates O_3_ production, accounting for up to 45.7% of HO_2_ generation within the CH_3_CHO degradation cascade [17]. However, atmospheric photochemical reactions evolve through a highly intricate network of pathways, influenced not only by local precursor intensities but also by meteorological forcing such as elevated temperatures, low humidity, and intense solar radiation, which collectively enhance the production of photo-oxidants including O_3_ [58]. Utilizing HCHO can provide a more precise and effective means of elucidating the mechanisms of indigenous photochemical pollution, especially in megacities with highly complex meteorological conditions.

Particularly, traditional PMF-derived source apportionment lacks the capacity to quantify the specific contributions of various meteorological drivers. Failure to account for meteorological influences may compromise the authenticity of source apportionment assessments in a complex atmospheric environment. In this study, a machine learning approach was employed to discern the nonlinear contributions of intricate meteorological features, biogenic and anthropogenic VOC emission sources to photochemical pollution. Considering that HCHO possesses complex nonlinear relationships with VOC precursors, and photochemical reactions entail certain temporal lags [30]. To reduce uncertainties caused by temporal–dynamic differences in the continuity of photochemical reactions and to resolve characteristic timescales, the reaction time correction methods were developed through systematic evaluating the influence of the factors with time lags ranging from 0 to 3 h during daytime hours (7:00–18:00). Summarized results in Table S6 indicated that the optimal fits for HCHO were achieved with a 1-h and 1.5-h lag at all three sites (R^2^ > 0.80), which also implied the photochemical reaction timescale may be between 60 and 90 min. Furthermore, the SHAP analysis was introduced to ascertain the driving contributions of the ten factors to HCHO generation through summary plots at the three sites (Figures S9–S11). Simultaneously, multi-model validation (XGBoost, LightGBM, RF) under identical 1-hour lag optimal conditions demonstrated consistent driving factor results, confirming XGBoost-derived HCHO output robustness while eliminating single-model bias (Figure S11). Among the models, XGBoost outperformed LightGBM and RF, achieving higher R2 values and lower RMSE, MSE, and MAE, as listed in Table S7. Furthermore, all three models demonstrated excellent fits across the three sites (R^2^ > 0.90), and the observed and predicted values were in satisfactory agreement under the optimal parameters, indicating that the models were sufficiently trained (Figure S12). In summary, HCHO demonstrated superior representativeness, and the XGBoost model effectively simulated the reaction mechanisms of the ten driving factors under photochemical conditions of the 1-hour time lag. All models were comprehensively trained and validated, providing reliable insights into the contributions of VOCs and meteorological factors to photochemical processes.

**Figure 4:**
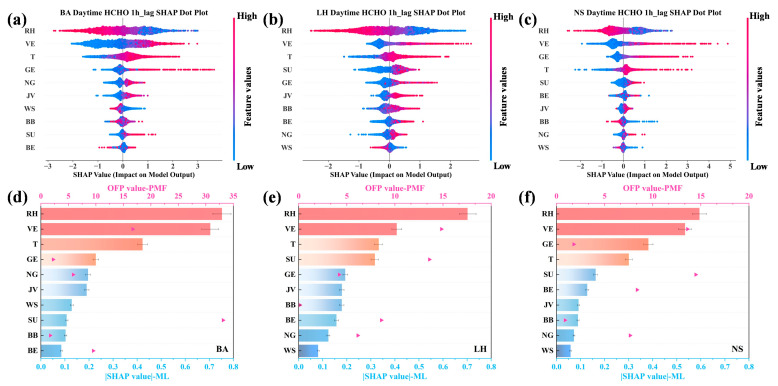
(**a**–**c**) The SHAP dot plots of HCHO. (**d**–**f**) The SHAP values and OFP from emission sources (RH = relative humidity; T = temperature; JV = photolysis rate; WS = wind speed; BB = biomass burning; VE = vehicle exhaust; GE = gasoline evaporation; NG = natural gas; SU = solvent usage; BE = background emissions).

Figure 4 presents the SHAP values of driving factors for HCHO generation under optimal test conditions, alongside comparisons of OFP. Meanwhile, all driving factors passed the variance inflation factor (VIF) test with high variable tolerance and no multicollinearity problem (VIF < 10, indicating negligible multicollinearity), further excluding the misleading effects of collinearity (Figure S13). As illustrated in Figure 4a–c, SHAP analysis revealed that RH and T played pivotal roles in HCHO generation, where lower humidity (blue dots) and higher temperatures (red dots) may have a positive correlation with HCHO production. This outcome is consistent with the frequent occurrence of high photochemical pollution under high-temperature and low-humidity meteorological conditions [51,54]. The JV exhibited a moderate contribution, suggesting a positive influence of solar radiation on the photochemical reactions driving atmospheric oxidant formation [59]. Conversely, the relatively low contribution of WS across the three sites reflected the limited effect of regional transport and lateral dispersion of pollution plumes, thus supporting the predominance of local emission sources. Among emission sources of VOCs, the concentrations of all factors were positively correlated with SHAP-derived HCHO values, reaffirming the significance of biogenic and anthropogenic emissions in driving HCHO generation, where higher concentrations of emissions were associated with greater impacts on HCHO production. In Figure 4d–f, the nonlinear perspective offered by the machine learning approach revealed a result that differs from the traditional linear view based on the OFP method. This contrast underscores the scientific validity and enhanced interpretative capacity of nonlinear approaches in elucidating the intricacies of atmospheric chemical processes. From this nonlinear perspective, VE emerged as the dominant anthropogenic source across all three sites during daytime hours, aligning with the high daytime activity of VE emissions in megacities, particularly near major roadways. Furthermore, VE emissions contribute to the production of active species such as HONO and OVOCs, which generate OH· and disrupt the NO_x_-O3 balance, enhancing HCHO formation and photochemical pollution [47,60]. This observation is consistent with previous regional studies [30], emphasizing the critical role of VE in driving urban photochemical processes during daytime. At the BA site, the heightened influence of GE and NG relative to SU, contrasting with the SU dominance indicated by PMF-derived OFP results, suggested the potential impact of GE and NG on HCHO generation [46,53]. From the perspective of LH, SU and GE were the principal active sources after VE due to the presence of numerous industrial parks and gas stations in the vicinity. VE and GE dominated the contributions at NS, which was a representative urban commercial district with high traffic density along major surrounding roads. Notably, the elevated contribution of GE at three sites highlighted the potential influence of gasoline evaporation, characterized by species such as alkanes, on HCHO generation. Moreover, the integrated emissions from VE and GE, which collectively constitute transportation-related sources in megacities, predominantly account for anthropogenic emissions as revealed through machine learning-based comprehensive assessments, which appear to substantiate the pivotal role of traffic-derived pollution in metropolitan atmospheric environments. While some features may influence HCHO indirectly through their effects on intermediate variables such as VOCs, the machine learning framework employed in this study is capable of capturing both direct and indirect relationships, which are reflected comprehensively in the model results.

**Figure 5:**
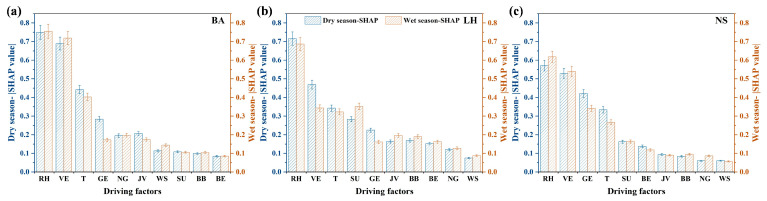
(**a**–**c**) The SHAP histograms of HCHO in wet and dry seasons at three sites (feature abbreviations are the same as in Figure 4).

To further reveal the mechanistic insights of the impact of driving factors under varying climatic conditions, the SHAP values were meticulously stratified into dry (spanning from November to April) and wet seasons (encompassing May to October), grounded in the recognition that Shenzhen is situated within a subtropical monsoon climate zone [61]. As depicted in Figure 5a–c, RH exerted a dominant role during the wet season, whereas T predominantly drove pollutant variations in the dry season, suggesting a climate sensitivity-dependent mechanism of meteorological factors. Supplementally, the atmospheric dynamics are governed by three distinct air masses in Shenzhen, including continental (northerly), marine (southerly), and coastal mixed air masses [62]. Seasonal shifts in air mass dominance are evident, where clean marine air masses (southern origin) prevail in the wet season, while polluted continental air masses from the north dominate the dry season [61]. Notably, although the spatial–seasonal disparities were continually underscored in regional pollution dynamics, anthropogenic emissions, especially the transportation sources, showed unapparent changes in the wet and dry seasons from nonlinear views by machine learning, which further highlighted the urgency and criticality of traffic atmospheric pollution in megacities. Meanwhile, the persistent VE dominance in megacities with high-density traffic and heterogeneous fleet profiles underscores unresolved challenges in electrification and emission standard enforcement. Contrarily, the traditional assessment method based on parameters may overlook or underestimate the impact of transportation sources while disproportionately amplifying contributions from short-lived photochemical sources (e.g., solvent usage and biogenic emissions).

## 4. Conclusions

Based on year-long VOC observations at three urban sites in Shenzhen, China, this study proposed an innovative diagnostic framework that integrates PMF with machine learning (XGBoost) and SHAP analysis to directly quantify the nonlinear contributions of VOC emissions to HCHO. Comprehensive observations revealed that OVOCs were the dominant VOCs. OVOCs, alkenes, and aromatics consistently contributed most to both OFP and OH· reactivity, with acetaldehyde, isoprene, and styrene identified as key reactive species influencing AOC. Using photochemical age-corrected VOCs data, PMF resolved six major sources, including solvent usage, vehicle exhaust, biogenic emission, natural gas, gasoline evaporation, and biomass burning. A novel reaction time correction framework was also established to address uncertainties in photochemical reaction timescales. SHAP analysis revealed that vehicle exhaust was the primary driver of HCHO formation, in contrast to traditional reactivity-based indicators (OFP), which tended to emphasize solvent use. This approach overcomes limitations of traditional indicators of VOC reactivity, such as OFP and *L*_OH_. Moreover, the increased importance of vehicle exhaust in HCHO formation revealed by the machine learning framework may be attributed to potential mechanisms such as the co-occurrence of HCHO with other oxidation-enhancing compounds near vehicular sources, and the Cl·-induced acceleration of alkane activation from vehicular emissions in coastal megacities. Overall, our findings underscore the dominant role of vehicular emissions in urban photochemical pollution, providing scientific evidence to support the integration of traffic planning with photochemical control strategies. These insights also contribute to mitigating public health risks associated with HCHO exposure in residential environments. While the SHAP-based analysis effectively captures nonlinear source–product relationships, future efforts will focus on incorporating causal inference to more robustly resolve the underlying chemical mechanisms.

## Data Availability

All relevant data are available in the main text.

## Supplementary Materials

The following supporting information can be downloaded at https://www.mdpi.com/article/10.3390/toxics13080643/s1. Text S1: Instrument and Analysis. Text S2: Instrument Principles and Quality Control. Text S3: Introduction of Photochemical Age Correction [19,20,21,22,32,33,34,35,63,64,65,66]. Text S4: Principles and Parameter Selection for PMF Analysis [19,36,37,39,40,67]. Text S5: Calculation of Ozone Formation Potential [41,42,45]. Text S6: Estimation of OH· Consumption Rate [41,63,64]. Text S7: The Framework of Machine Learning and Bayesian Optimization [24,27,28,29,30,31,43]. Text S8: The Overview of Meteorological Factors. Figure S1: (a–c) The relationship between the concentration of m,p-Xylene and ethylbenzene at three sites; (d–f) the diurnal of the X/E at three sites. Figure S2: The time series of (a) concentrations of total measured VOCs (TVOCs), (b) temperature (°C), (c) relative humidity (%), and (d) wind speed (m/s) at three sites. Figure S3: Annual time series of VOC components percentage at (a) BA, (b) LH, and (c) NS. Figure S4: Solar maps of 116 VOC species concentrations and components at BA, LH, and NS. Figure S5: Ozone formation potential ranking and proportion of OH· consumption of VOC species. Figure S6: The PMF profiles of (a) BA, (b) LH, and (c) NS. Figure S7: The natural gas source profiles of (a) offline samples, (b) BA, (c) LH, and (d) NS. Figure S8: The SHAP model of HCHO output at different time lags (0–3 h) under the influence of multiple factors by XGBoost at BA. Figure S9: The SHAP model of HCHO output at different time lags (0–3 h) under the influence of multiple factors by XGBoost at LH. Figure S10: The SHAP model of HCHO output at different time lags (0–3 h) under the influence of multiple factors by XGBoost at NS. Figure S11: The SHAP model of HCHO output at 1 h lag under the influence of multiple factors by XGBoost, LightGBM, and Random Forest. Figure S12: Scatter plots of observation values from PMF and prediction values from ML at three sites under (a–c) XGBoost, (d–f) LightGBM, (g–i) RF, and (j–l) the time series of prediction and observation by XGBoost and PMF at three sites. Figure S13: Heatmap and bar charts of variance inflation factor (VIF) between multiple factors output from the SHAP values. Table S1: The information of the monitoring sites. Table S2: The concentration levels of monitoring species. Table S3: The list of representative species for PMF. Table S4: The test results of PMF. Table S5: Ratio results of key species using the characteristic ratio method. Table S6: Machine learning outputs test results at various intervals for HCHO by XGBoost at three sites. Table S7: Machine learning outputs test results under optimal conditions by various models at three sites.
