# Supplementary material for: Decoding the Primacy of Transportation Emissions of Formaldehyde Pollution in an Urban Atmosphere
Source: Toxics. 2025 Jul 30;13(8):643. doi: 10.3390/toxics13080643 (PMC12390000; doi:10.3390/toxics13080643)
Supplement: Supplementary file 1 [file toxics-13-00643-s001.zip › toxics-3756371-supplementary.pdf]

Supporting Information

Decoding the Primacy of Transportation Emissions of Formaldehyde Pollution in an Urban Atmosphere

Shi-Qi Liu [1,†], Hao-Nan Ma [1,†], Meng-Xue Tang [1,*], Yu-Ming Shao [1], Ting-Ting Yao [2], Ling-Yan He [1] and Xiao-Feng Huang [1]

[1] Key Laboratory for Urban Habitat Environmental Science and Technology, Peking University Shenzhen Graduate School, Shenzhen 518055, China

[2] Shenzhen Academy of Metrology and Quality Inspection, Shenzhen 518107, China

* Correspondence: tangmx@pku.edu.cn

† These authors contributed equally to this work.

Supplementary Text

Text S1: Instrument and Analysis

VOCs were quantified using the ZF-PKU-VOC1007 continuous online monitoring system (Beijing Pengyuchangya, China), which combined an electric refrigeration cryogenic trapping system with a detection apparatus comprising a GC-MS system (GCMS 8860-5977B, Agilent, USA). This sophisticated system enabled detection with hourly resolution for quantifying 116 VOC species, including 29 alkanes, 12 alkenes, 17 aromatics, 34 halohydrocarbons, 21 oxygenated VOCs (OVOCs), and additional compounds such as acetylene, carbon disulfide, and acetonitrile.

Formaldehyde (HCHO) was monitored through the FMS-100 online formaldehyde monitoring system (Hangzhou Juguang, China). This instrument amalgamated modular components for gas sampling, chemical reaction (via Hantzsch derivatization), signal conversion, flow control, zero gas trap, and an industrial control computer module. Quantitative detection of atmospheric HCHO was achieved through fluorescence signals detected and converted by the photomultiplier tube (PMT), achieving 5-minute temporal resolution.

Meteorological parameters (temperature [T], relative humidity [RH], wind speed [WS]) were measured using the HCD6815 multi-parameter integrated sensor array (Hebei Yunnong, China) with 10-minute resolution. The device simultaneously measured

atmospheric pressure, wind direction, and dew point temperature. Photolysis frequencies (JV) were specifically determined using the PFS-100 atmospheric photolysis rate analyzer (Hangzhou Juguang, China), capable of online and continuous measurements of photolysis rates for various atmospheric species (e.g., O_1D, NO_2, NO_3, HONO, HCHO, H_2O_2). In this study, the photolysis rate of NO_2 (JNO_2) was primarily represented for JV with a time resolution of approximately 10 seconds.

All aforementioned instruments were collocated at a single station, ensuring synchronized continuous observations characterized by high consistency and data reliability.

Text S2: Instrument Principles and Quality Control

At three monitoring sites, the ZF-PKU-VOC1007 performed cyclic sampling operations with hourly intervals each day. A cryogenic enrichment system was implemented to capture VOCs from ambient air and the sampling inlet was outfitted with particulate-removal filter membranes. Teflon-lined tubing featuring integrated thermal regulation prevented moisture condensation and aerosol deposition, ensuring measurement fidelity. Collected samples were introduced into the gas chromatography-mass spectrometry (GC-MS) system and bifurcated into two distinct detection pathways: a flame ionization detector (FID) and a mass spectrometer (MS). The FID pathway was responsible for detecting thirteen low-carbon hydrocarbons (C_2-C_5), while the MS pathway identified

103 species including hydrocarbons (C_5-C_{12}), halogenated hydrocarbons, OVOCs and acetonitrile. For the FID pathway, the gas stream traversed quartz water-removal tubes and CO_2-removal tubes filled with lime granules to eliminate water vapor and carbon dioxide. Subsequently, the sample was cryogenically concentrated using a PLOT (Porous Layer Open Tubular) Al_2O_3/KCl capillary column trap (0.53 mm inner diameter, -155 ℃). In the MS pathway, the gas passed through a quartz water-removal tube maintained at -10 ℃ before undergoing cryogenic concentration in a deactivated quartz capillary trap at -155 ℃. Following cryogenic trapping, samples were thermally desorbed at 120 ℃ and analyzed by the GC-MS/FID system. In the FID pathway, gases were chromatographically separated using a PLOT (Al_2O_3/KCl) column and quantitatively analyzed by the FID detector, utilizing helium as the carrier gas. Conversely, in the MS pathway, gases were separated using a DB-624 mid-polarity chromatographic column before being directly introduced into the MS detector. Post-detection, high-purity nitrogen gas was employed for thermal back-flushing at 120 ℃ to purge residual impurities from the system.

Instrument calibration underwent monthly calibration protocols with certified reference gases (Linde Gas, USA) spanning five concentration levels. Calibration curves for over 90% of the detected species achieved coefficients of determination (R^2) exceeding 0.98. Quantification via the FID was performed using external standards (PAMS, TO-15, and aldehyde/ketone standards), while MS quantification utilized internal standards comprising bromochloromethane, 1-bromo-4-fluorobenzene, chlorobenzene-d_5, and 1,4-

dichlorobenzene. Method detection limits (*MDL*) were established at 0.006-0.074 ppb through serial dilution studies. Instrument performance was validated daily by midnight (at 0:00) calibration checks using median point from five concentration levels, ensuring continuous measurement precision and data reliability.

The formaldehyde online monitoring system (FMS-100) collected atmospheric samples by the vacuum pump connected through PTFE tubing (6.0 mm). HCHO was absorbed into the internal H_2SO_4 solution (analytical grade, Merck) and subsequently reacted with Hantzsch reagent (analytical grade, Merck) within a temperature-controlled reaction chamber to form 3,5-diacetyl-1,4-dihydro-2,6-dimethylpyridine (DDL). As a fluorescent product, DDL absorbed ultraviolet light at 410 nm and emits fluorescence at 510 nm, which was detected by PMT to quantify HCHO concentrations. Due to the instability of HCHO calibration gases, calibration was performed employing HCHO standard solutions. Quality control protocols included: weekly single-point standard solution assessments, automated baseline calibrations every 12 hours, fortnightly two-point checks, and monthly five-point calibrations. Calibration curves demonstrated consistent linearity with R^2 values exceeding 0.99. The instrument achieved a detection limit of 0.05 ppb with a measurement range spanning from 0.05 to 3000 ppb, enabling precise trace-level analysis.

The multi-parameter integrated sensor array meteorological instrument (HCD6815) incorporated ultrasonic probes, control circuits, and an integrated louvered shield. Within

the shield, an array of sensors continuously recorded temperature, relative humidity, wind speed/direction, and atmospheric pressure. Signals from these sensors were processed by the built-in microprocessor which amplified, filtered, and digitized the data to obtain accurate meteorological parameters. The high integration of instrument and stability rendered it suitable for long-term field observations.

The atmospheric photolysis rate analyzer (PFS-100) comprised a quartz optical receiver, a spectrometer, an industrial control computer, and fiber-optic transmission lines. The receiver collected solar radiation from all directions and transmitted them via quartz optical fiber to the spectrometer. The spectrometer captured spectral data within the 270-790 nm range and relayed the information to the industrial control computer. The computer converted the spectral signals into photochemical flux $F(\lambda)$, and photolysis rate constants were calculated by integrating $F(\lambda)$ with the known absorption cross-section $\sigma(\lambda)$ and quantum yield $\Phi(\lambda)$. Routine maintenance of the PFS-100 included fortnightly optical receiver surface cleaning with anhydrous ethanol and monthly desiccant replacement in the drying tube of receiver.

Text S3: Introduction of Photochemical Age Correction

VOCs undergo depletion not only through atmospheric diffusion and deposition but also via photochemical oxidation reactions with hydroxyl radicals (OH·), O_3, and other reactive species under photolytic conditions. Neglecting this photochemical degradation

and directly utilizing observed concentration data from monitoring sites could lead to inaccuracies and systematic biases in source composition analysis and VOC pollution assessments.[1,2] To address this, photochemical age correction was calculated to adjust VOC concentrations for photochemical losses, thereby deriving initial emission concentrations. The mathematical framework for this correction is described in Equations 1-2:[1,3,4]

$$[VOC_i]_{inital} = [VOC_i]_{detected} \times e^{(k_i \times [OH]\Delta t)} \quad (1)$$

$$[OH]\Delta t = \frac{1}{k_X - k_E} \times \left(ln\left(\frac{[X]}{[E]}\right)_0 - ln\left(\frac{[X]}{[E]}\right)_t \right) \quad (2)$$

$[VOC_i]_{inital}$ and $[VOC_i]_{detected}$ represent the corrected initial concentration and the observed concentration (ppb) of different VOC species, respectively. k_i represents the rate constant of the OH· for each VOC species ($cm^3 \cdot molecule^{-1} \cdot s^{-1}$), which varies across species.[5,6] $[OH]$ denotes the average atmospheric concentration of OH· ($molecules \cdot cm^{-3}$), and Δt represents the photochemical age of the air mass (s). The product $[OH]\Delta t$, often referred to as the OH· exposure, can be calculated using the ratio of two VOC species with different reaction rate constants but similar emission sources.[7] In this study, the ratio of m,p-xylene (k_X = 18.7×10^{-12} $cm^3 \cdot molecule^{-1} \cdot s^{-1}$) to ethylbenzene ($k_E$ = 7.0×10^{-12} $cm^3 \cdot molecule^{-1} \cdot s^{-1}$) was utilized for estimation, which these two species demonstrated strong correlations across the three monitoring sites ($R^2 > 0.90$, $p < 0.05$) (Figure S1a-c). $\left(\frac{[X]}{[E]}\right)_0$ as the initial concentration ratio represents the ratio of these species prior to the aging of the air mass and was determined as the maximum value during non-photolytic

nighttime hours (0:00-6:00) to account for nighttime VOC-NO_2 reactions.[2] The initial ratios at the three sites are depicted in Figure S1d-f, while $\left(\frac{[X]}{[E]}\right)_t$ denotes the concentration ratio during the measurement period. Although this method presents limitations in calculating OH· exposure such as its inability to account for uncertainties arising from the mixing of fresh and aged air masses that alter VOC compositions and error in temperature dependence,[8,9] it is widely recognized as an effective approach for evaluating photochemical losses.[10]

Nevertheless, isoprene, which is a predominant biogenic volatile organic compound (BVOC) emitted by urban vegetation, exhibits high sensitivity to temperature and light intensity with emissions peaking at midday.[11] Employing the X/E ratio method for isoprene may lead to substantial overestimation of concentrations due to its rapid oxidation. Consequently, the initial concentration of isoprene was reconstructed using its photochemical oxidation products such as methacrolein (MACR) and methyl vinyl ketone (MVK) based on the reaction relationships (Equation 3), with specific details provided in Equations 3-6:[12]

$$OH \cdot + Isoprene \rightarrow 0.63HCHO + 0.23MACR + 0.32MVK \tag{3}$$

$$\frac{[MACR]_t}{[Isoprene]_t} = \left(1 - e^{(k_1 - k_2)[OH]\Delta t}\right) \times \frac{0.23k_1}{k_2 - k_1} \tag{4}$$

$$\frac{[MVK]_t}{[Isoprene]_t} = \left(1 - e^{(k_1 - k_3)[OH]\Delta t}\right) \times \frac{0.32k_1}{k_3 - k_1} \tag{5}$$

$$[Isoprene]_{initial} = [Isoprene]_t \times e^{(k_1 \times [OH] \Delta t)} \tag{6}$$

In the equations, $[MACR]_t$, $[MVK]_t$, and$[Isoprene]_t$ denote measured concentrations (ppb) of MACR, MVK and isoprene, respectively. $[Isoprene]_{initial}$ represents the initial concentration of isoprene after photochemical age correction (ppb). The OH· reaction rate constants of isoprene, MACR and MVK are defined as k_1, k_2, and k_3, respectively.[5,12]

Text S4: Principles and Parameter Selection for PMF Analysis

The methodological bilinear framework is based on the principles delineated[13] as shown in Equations 7-8:

$$x_{ij} = \sum_{k=1}^{p} g_{ik} f_{kj} + e_{ij} \tag{7}$$

$$Q = \sum_{i=1}^{n} \sum_{j=1}^{m} \left(\frac{e_{ij}}{u_{ij}} \right)^2 \tag{8}$$

Which x_{ij} represents the concentration of the j species in the i sample; g_{ik} is the contribution of the k source to the i sample, denoting the source contribution; f_{kj} is the source factor profile, indicating the concentration contribution of the j species in the k source; e_{ij} is the residual of the model; and u_{ij} is the estimated uncertainty of the j species in the i sample. The objective function Q represents the model iteration target and is a critical parameter for determining model stability, optimal results of the factorization are achieved when Q reaches its minimum value.[14,15]

Uncertainties (Unc) were calculated based on the method detection limits (MDL) of each species, following the procedures outlined in the PMF 5.0 User Guide.[16] The calculations adhere to the equations below (Equation 9-10):

$$Unc = \sqrt{(Error\ Fraction \times con.)^2 + (0.5 \times MDL)^2} \quad con > MDL \tag{9}$$

$$Unc = {}^{5}/_{6} \times MDL \quad 0 < con \leq MDL \tag{10}$$

The Error Fraction (EF) values are typically constrained within a reference range of 5-30%. In this study, the EF assignments for each species was determined according to the OH· reaction rate constants (K_{OH}) as follows: $K_{OH} \leq 5 \times 10^{-12}$ cm^3 molecule^{-1} s^{-1}, EF = 10%; 5×10^{-12} cm^3 molecule^{-1} s^{-1} < $K_{OH} \leq 50 \times 10^{-12}$ cm^3 molecule^{-1} s^{-1}, EF = 20%; K_{OH} > 50×10^{-12} cm^3 molecule^{-1} s^{-1}, EF = 30% (Table S3). Additionally, the concentrations of retained species were categorized based on their signal-to-noise (S/N) ratios following the PMF 5.0 User Guide. Specifically, $S/N > 2.0$ was classified as strong, $0.5 < S/N < 2.0$ as weak, and $S/N < 0.5$ as bad.[2,16]

The optimal number of pollution sources (k) was determined by testing 4-7 factor solutions and evaluating the Q_{true}/Q_{exp} ratio. Model robustness was further validated using bootstrapping (BS) and displacement (DISP) methods (Table S4). Results demonstrated that the most substantial variations in the Q_{true}/Q_{exp} ratio occurred when five factors were selected as the output at all three sites. Moreover, the DISP analysis revealed no source swaps indicating the absence of tailing effects, and the BS mapping

results for all factors exceeded 80%, thereby meeting the recommended threshold outlined in the PMF 5.0 User Guide.[17] Consequently, the model fitting outcomes in this study exhibited the operational reliability and scientific validity.

Text S5: Calculation of Ozone Formation Potential

The ozone formation potential (OFP) serves as a pivotal metric for assessing the contribution of VOCs to O_3 synthesis. After computing the OFP based on photochemical age correction of initial VOC concentrations, a more exhaustive evaluation of VOC quantitative impact on O_3 formation is attainable. The calculation is delineated in Equation 11:

$$OFP_i = [VOC]_{initial} \times MIR_i \tag{11}$$

Herein, the maximum incremental reactivity (MIR_i) is defined as the change in O_3 production per unit VOC mass (g O_3/g VOC) in the given air mass.[18] OFP_i denotes the photochemically corrected initial ozone formation potential of species i (ppb), while $[VOC]_{initial}$ represents the corrected initial concentration of species i (ppb). Importantly, it is imperative to acknowledge that the estimation of OFP is inherently subject to uncertainties in MIR values. These uncertainties stem from variations in chemical reaction mechanisms, regional meteorological conditions, and the overall atmospheric composition. Although it is recognized that some degree of error may persist, MIR values were rigorously selected based on an extensive review of multiple studies conducted by

Carter.[19,20]

Text S6: Estimation of OH· Consumption Rate

To enhance the quantitative assessment of VOCs as essential photochemical-reaction precursors, reaction consumption rate with OH· were incorporated, which are the dominant daytime oxidant and contribute more than 80% of the global daytime photochemical reactions.[5,6] Quantifying the consumption rate of OH· by VOCs provides a more holistic comprehension of the influence of initial VOC concentrations on atmospheric oxidation processes and their consequent contributions to photochemical pollution mechanisms. The methodologies for calculating the consumption rate of OH· for each VOC species are articulated in Equations 12:

$$L_{OHi} = [VOC]_i \times K_{OHi} \tag{12}$$

In these equations, L_{OHi} denotes the consumption rate of OH· by VOC species i (s^{-1}); $[VOC]_i$ represents the concentration of VOC species i (ppb); K_{OHi} is the reaction rate constant of OH· with species i (cm^3 $molecule^{-1}$ s^{-1}).[5,6,20]

Text S7: The Framework of Machine Learning and Bayesian Optimization

The XGBoost (Extreme Gradient Boosting) model, which is regarded as an advanced algorithm through additive tree ensemble construction, aims to minimize the objective function $\mathcal{L}(\phi)$, which is mathematically defined as in Equation 13:[21,22]

$$\mathcal{L}(\phi) = \sum_{i=1}^{n} l(y_i, \widehat{y_i}) + \sum_{k=1}^{K} \Omega(f_k) \tag{13}$$

In this formulation, $l(y_i, \widehat{y_i})$ denotes the mean squared error (*MSE*) loss function, quantifying the discrepancy between the predicted value $\widehat{y_i}$ and the true value y_i for the i-th sample; $\Omega(f_k)$ represents the regularization component, which modulates model complexity to mitigate overfitting and enhance generalization capabilities.[21,23] Within meteorological and environmental pollution research, the XGBoost model has gained prominence due to its remarkable efficiency and effectiveness.[24,25]

Shapley Additive Explanation (SHAP) is an interpretable framework for machine learning models grounded in Shapley values derived from cooperative game theory. It is designed to quantify the contribution of each feature to the overall model output.[26] The SHAP value ϕ_i for feature x_i is shown in Equation 14:[26]

$$\phi_i = \sum_{S \subseteq \{1,2,\ldots,M\} \setminus \{i\}} \frac{|S|!\,(M - |S| - 1)!}{M!} [f(S \cup \{i\}) - f(S)] \tag{14}$$

Herein, $S \subseteq \{1, 2, \ldots, M\} \setminus \{i\}$ encompasses all possible subsets of features excluding x_i, and the weight $\frac{|S|!(M-|S|-1)!}{M!}$ ensures that the contribution of feature x_i is equitably distributed across all possible feature combinations. $f(S \cup \{i\}) - f(S)$ reflects the marginal contribution of feature x_i upon its inclusion in subset S. After training machine learning models, the SHAP methodology adeptly quantifies the nonlinear contributions of diverse independent variables to the predictions of the model.[22,24]

Hyperparameter optimization is a critical problem in machine learning model construction. Conventional grid search techniques fail to incorporate prior parameter information, resulting in excessive computational costs. In contrast, Bayesian optimization iteratively augments the posterior distribution of the optimization function by incorporating new sample points, thereby leveraging prior information to achieve efficient hyperparameter tuning with reduced iterations, accelerated convergence, and mitigated risks of dimensionality explosion.[27] A Gaussian Process (GP) was used as a surrogate model within Bayesian optimization, assuming that the objective function $f(x)$ follows to a multivariate normal distribution (Equation 15):

$$f(x) \sim \mathcal{N}\big(\mu(x), \kappa(x, x')\big) \tag{15}$$

Given a dataset $\mathcal{D}_t$, the posterior distribution of the Gaussian process is expressed as in Equation 16:

$$f(x^*) \mid \mathcal{D}_t \sim \mathcal{N}\big(\mu^*(x), \sigma^2(x)\big) \tag{16}$$

In Equation 17, the fundamental process of Bayesian optimization is articulated:

$$x_{t+1} = arg \max_{x \in \mathcal{X}} \alpha\,(x; \mathcal{D}_t) \tag{17}$$

The optimization strategy initiates with a set of initial points x, from which outputs are derived via the objective function, thereby establishing the initial dataset $\mathcal{D}_0$. The Gaussian process is then fitted using the existing data $\mathcal{D}_t$. Within the parameter space $\mathcal{X}$, the

objective is to identify a point x_{t+1} that maximizes the acquisition function $\alpha(x;\mathcal{D}_t)$.The objective function $f(x)$ is subsequently evaluated at this point to obtain $f(x_{t+1})$, and the new point $\left(x_{t+1}, f(x_{t+1})\right)$ is incorporated into the dataset $\mathcal{D}_{t+1}$.

Text S8: The Overview of Meteorological Factors

From a meteorological standpoint (Figure S2b-d), the BA site recorded annual average values of temperature (25.9 ± 5.9 °C), relative humidity (76.6 ± 14.2%), wind speed (0.63 ± 0.35 m/s) and photolysis rate of JNO_2 (1.7 ± 1.5×10^{-3} s^{-1}). The LH site exhibited 25.5 ± 6.2 °C, 74.3 ± 15.0%, 1.96 ± 0.84 m/s, and 3.1 ± 2.3×10^{-3} s^{-1}, respectively, while NS exhibited 26.3 ± 5.7 °C, 72.6 ± 13.9%, 1.76 ± 0.79 m/s, and 3.3 ± 2.4×10^{-3} s^{-1}. The BA site experienced lower photolysis rates and wind speeds, resulting in suppressed photochemical reactions and limited pollutant dispersion, thus favoring pollutant accumulation influenced by local anthropogenic emissions. On the contrary, elevated photolysis rates and wind speeds at LH and NS accelerated more vigorously photochemical activities and dispersion of VOCs, implying that these sites could encounter secondary VOC formation and pollutant transport from external regions.

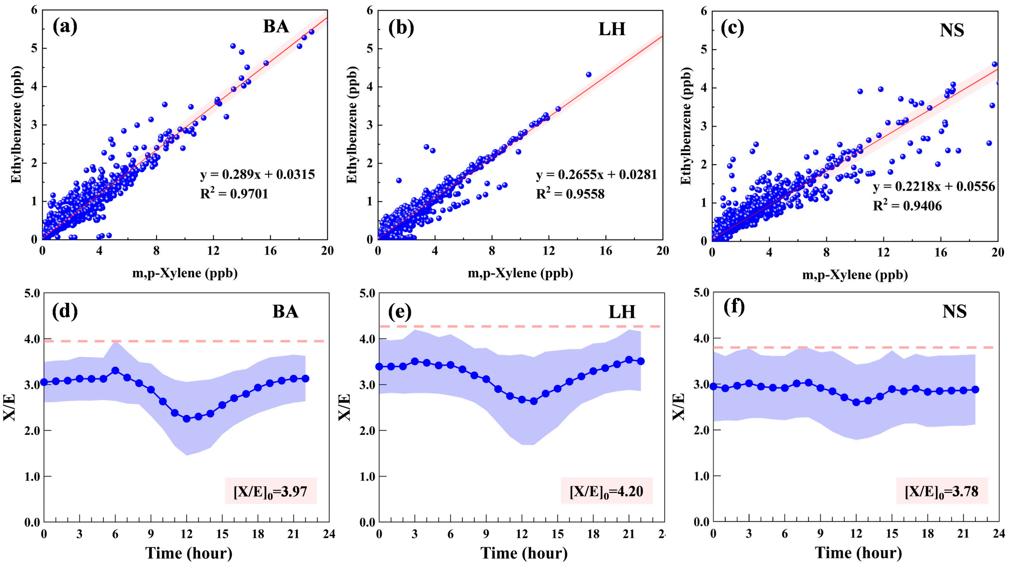

Figure S1. (a-c) The relationship between the concentration of m,p-Xylene and ethylbenzene at three sites; (d-f) The diurnal of the X/E at three sites.

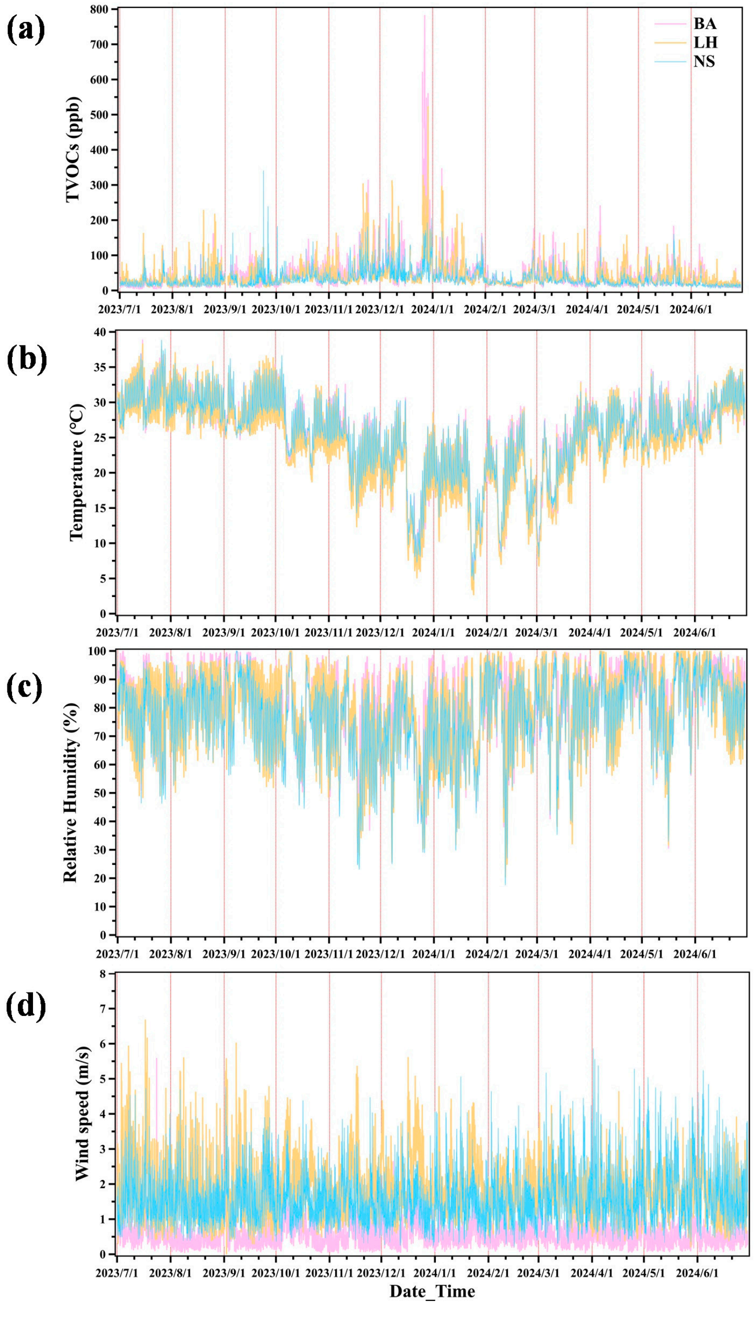

Figure S2. The time series of (a) concentrations of total measured VOCs (TVOCs); (b) temperature (°C); (c) relative humidity (%); (d) wind speed (m/s) at three sites.

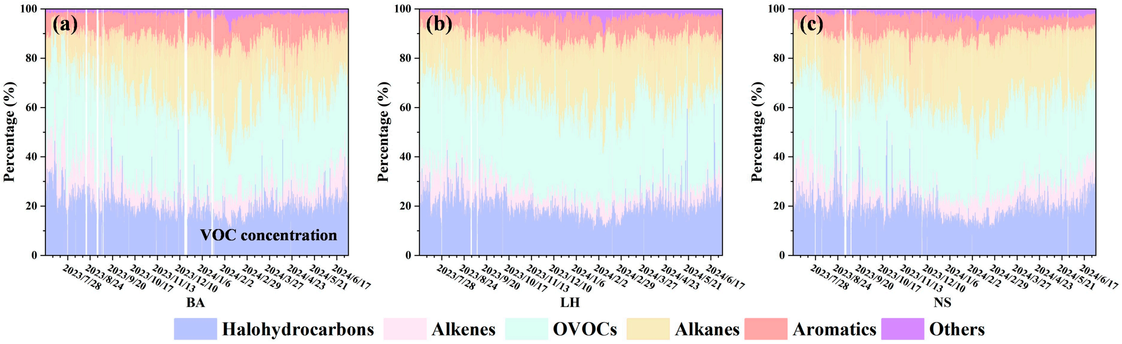

Figure S3. Annual time series of VOC components percentage at (a) BA; (b) LH; (c) NS

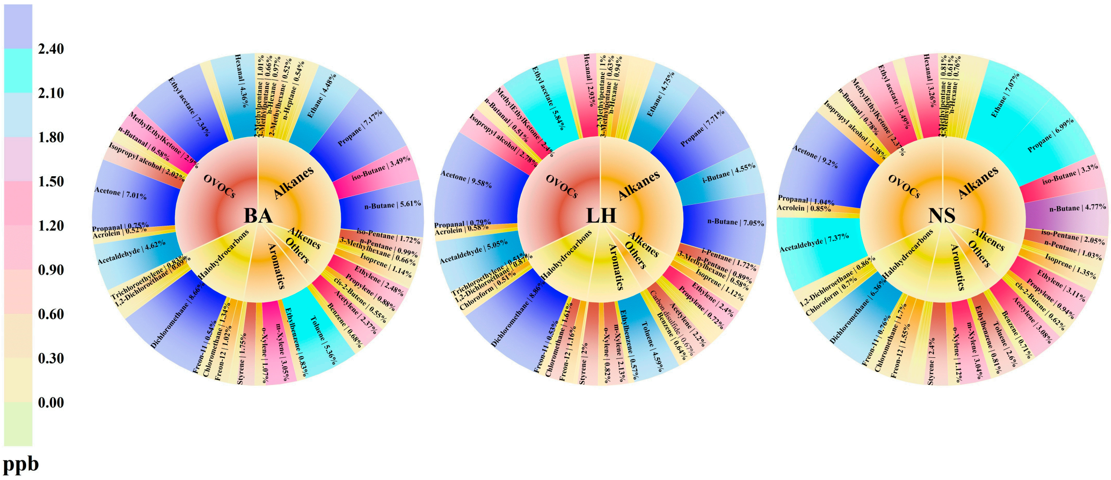

Figure S4. Solar maps of 116 VOC species concentrations and components at BA, LH and NS.

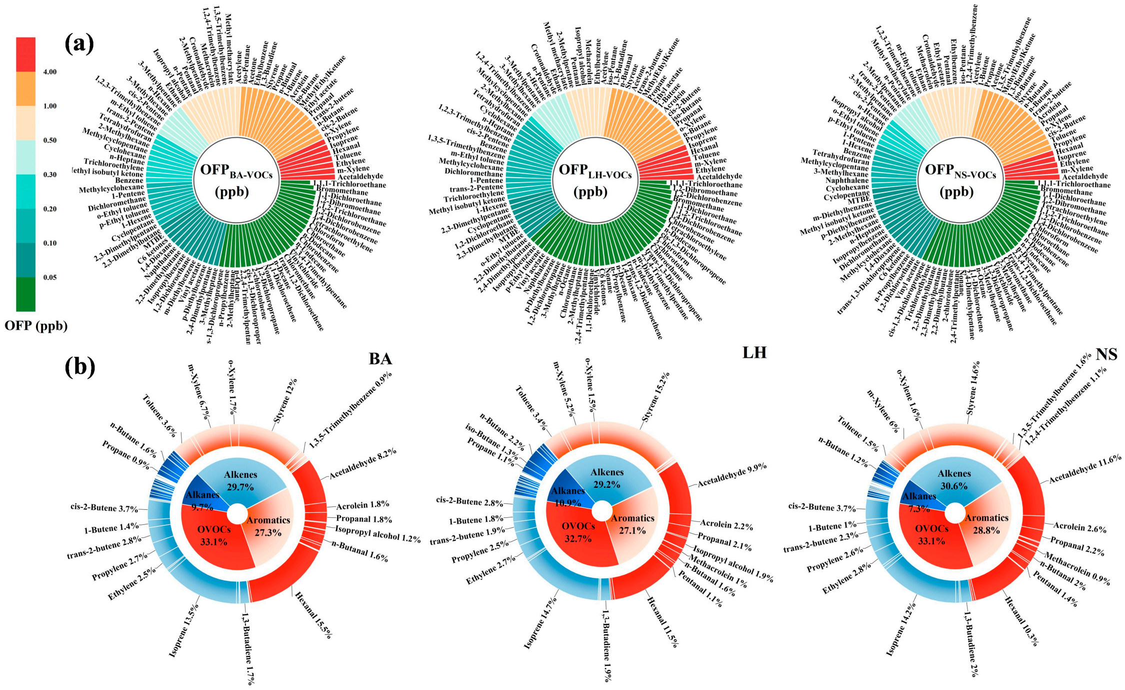

Figure S5. Ozone formation potential ranking and proportion of OH· consumption of VOC species

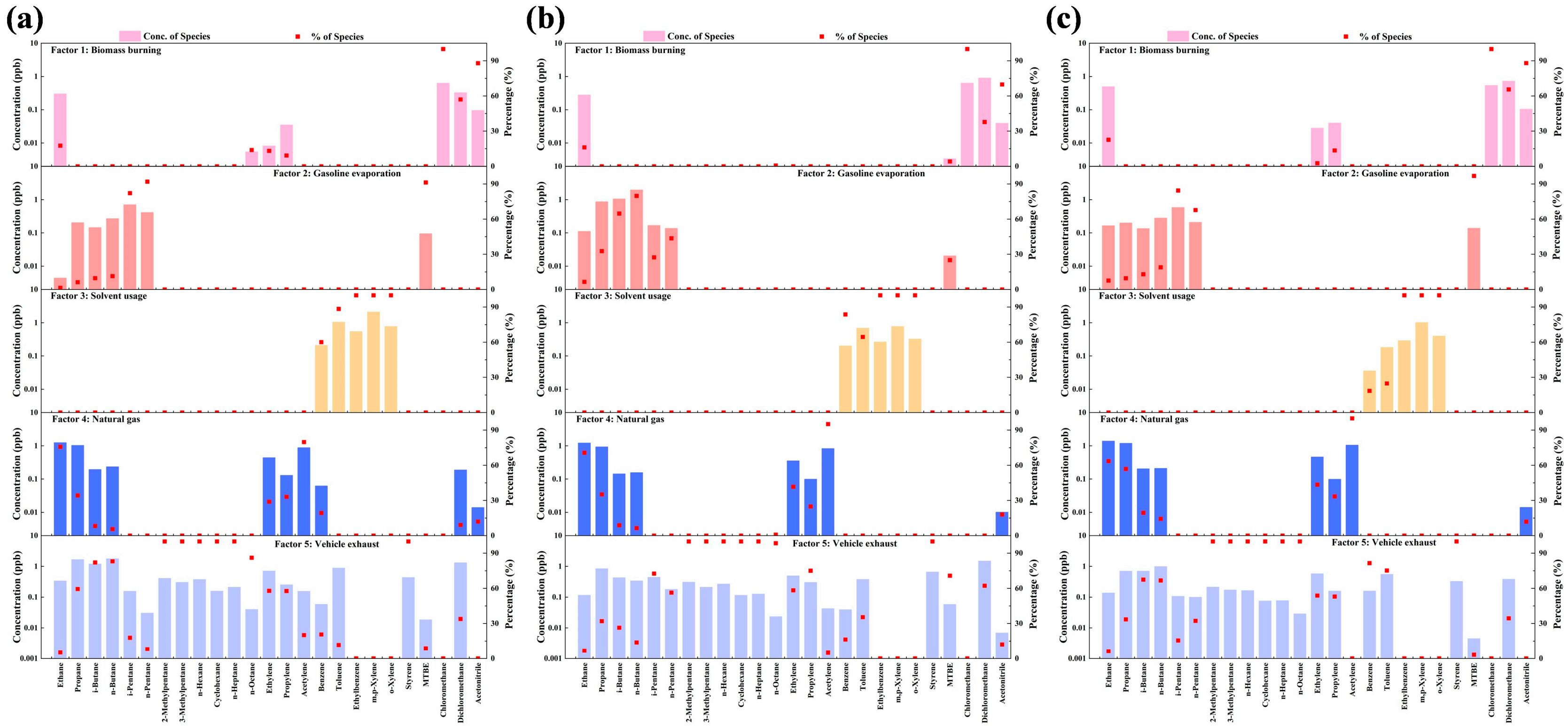

Figure S6. The PMF profiles of (a) BA; (b) LH; (c) NS.

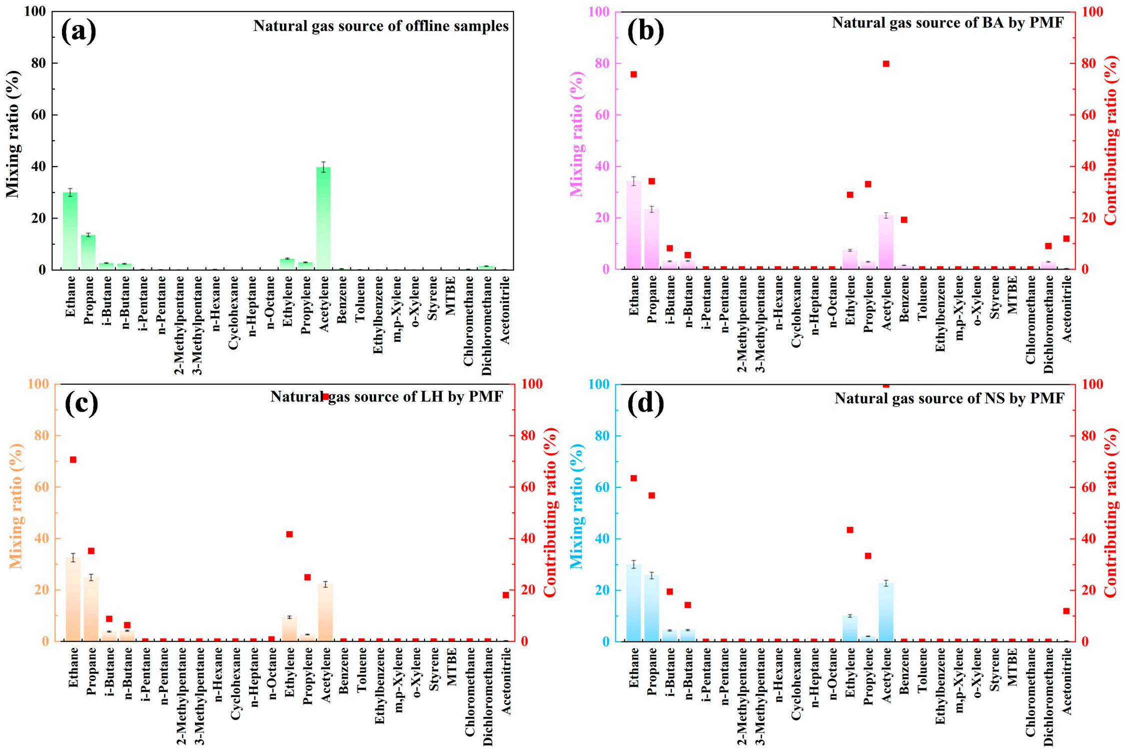

Figure S7. The natural gas source profiles of (a) offline samples; (b) BA; (c) LH; (d) NS.

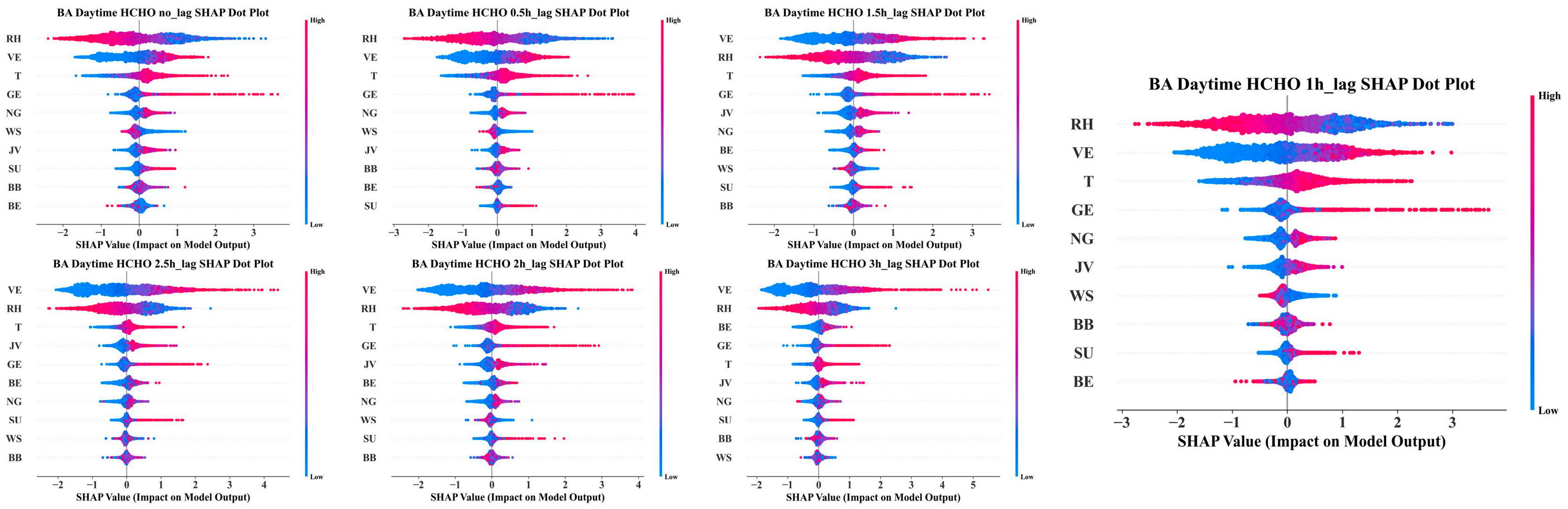

Figure S8. The SHAP model of HCHO output at different time lags (0-3 h) under the influence of multiple factors by XGBoost at BA.

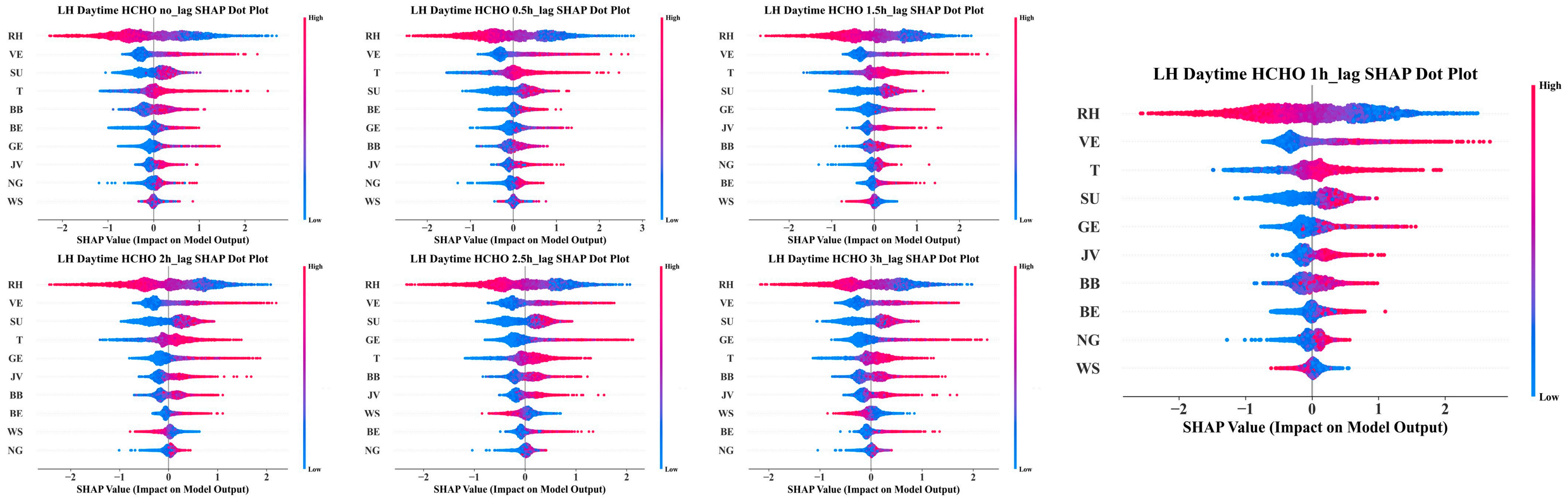

Figure S9. The SHAP model of HCHO output at different time lags (0-3 h) under the influence of multiple factors by XGBoost at LH.

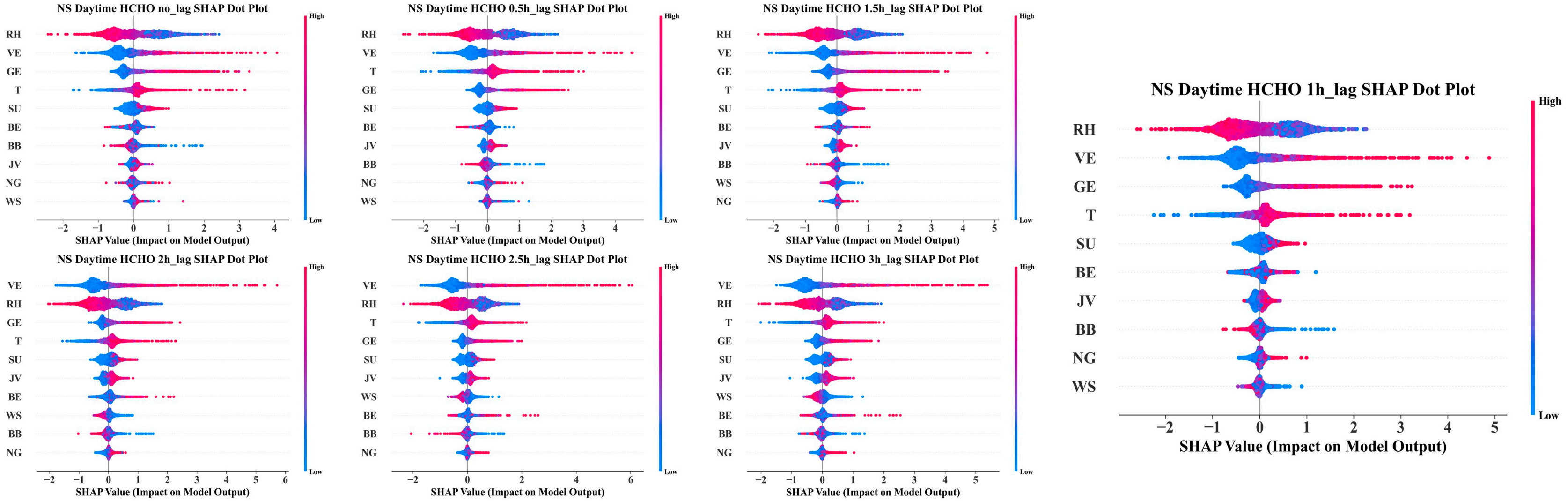

Figure S10. The SHAP model of HCHO output at different time lags (0-3 h) under the influence of multiple factors by XGBoost at NS.

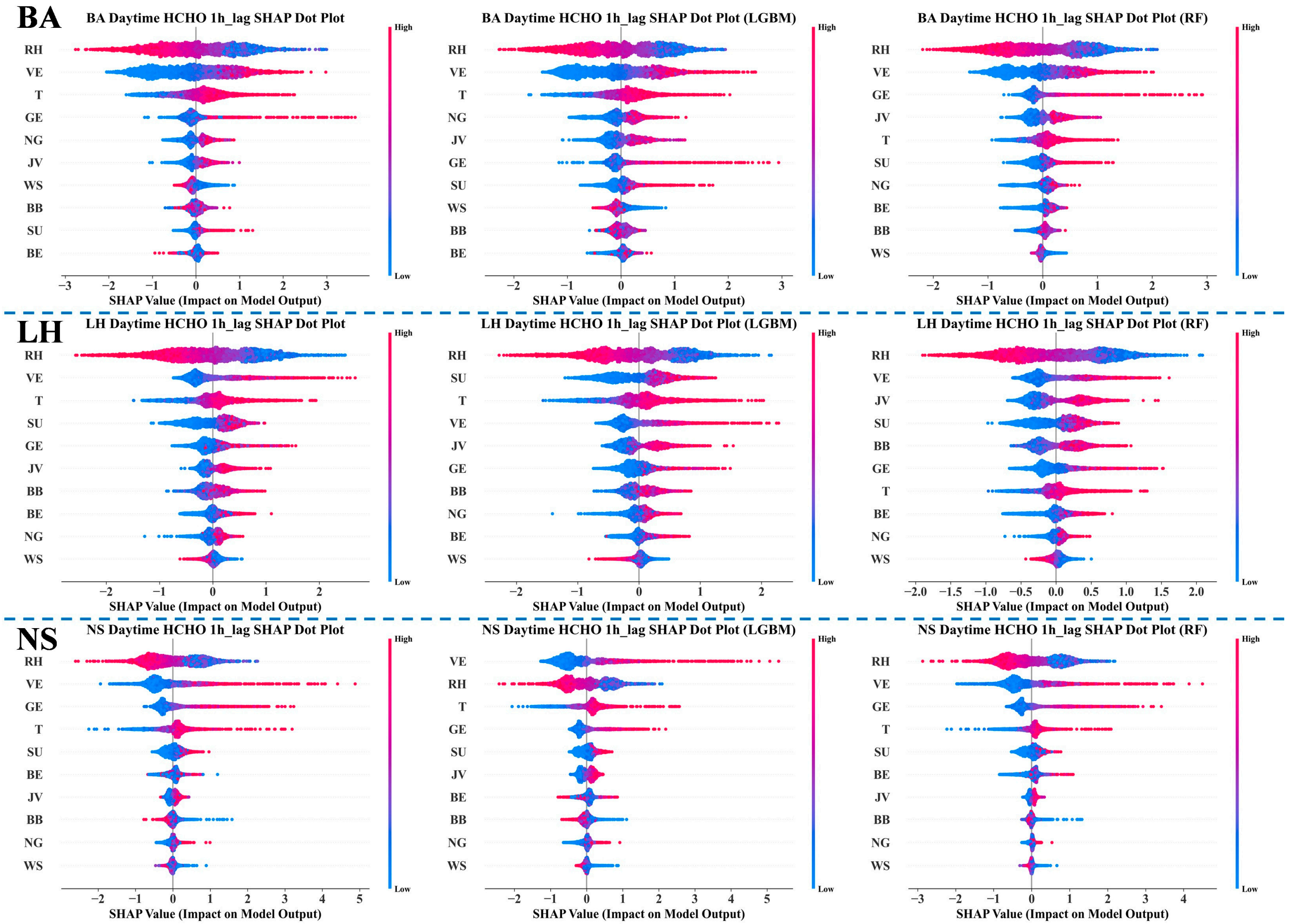

Figure S11. The SHAP model of HCHO output at 1 h lag under the influence of multiple factors by XGBoost, LightGBM and Random Forest.

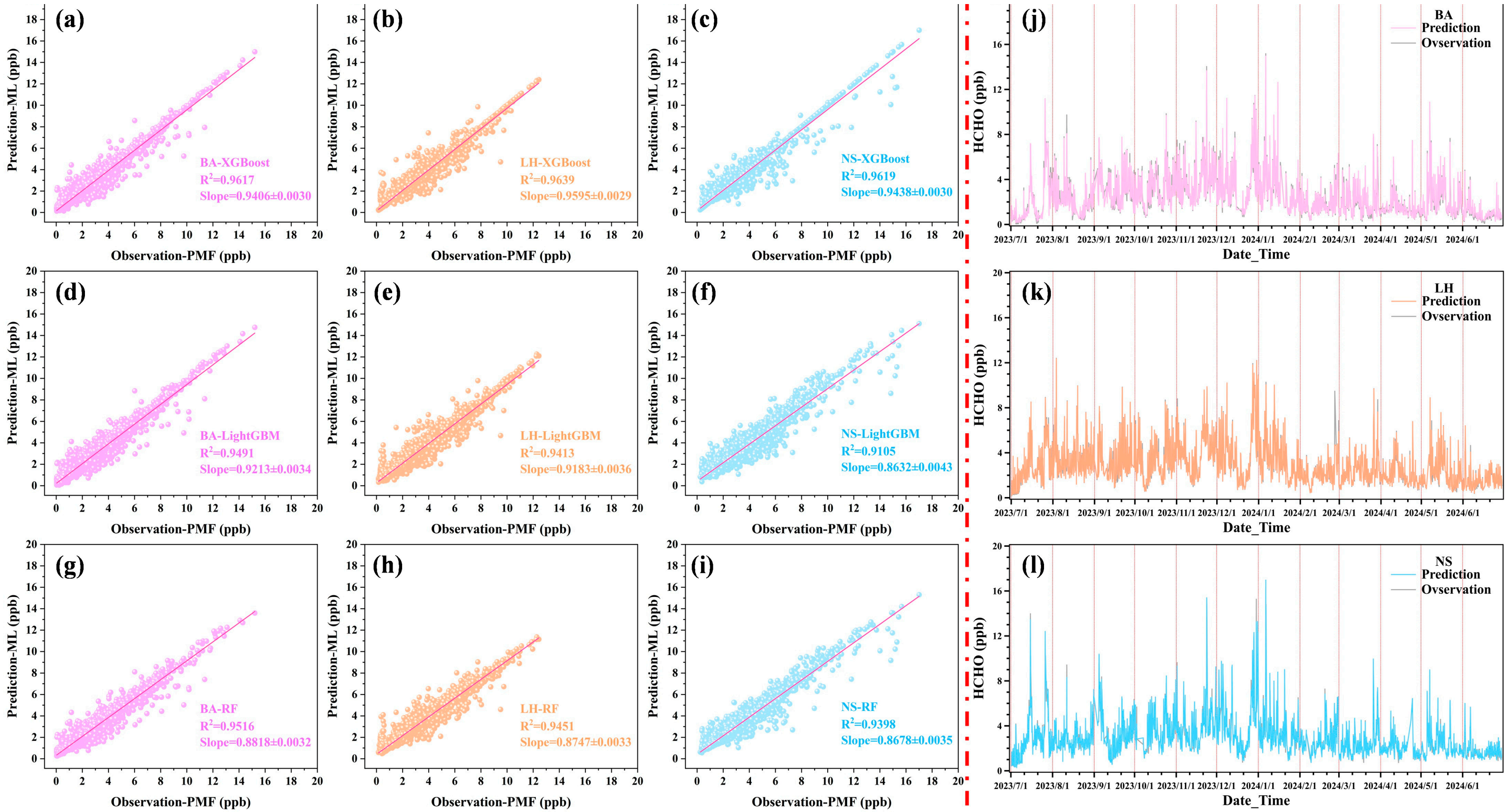

Figure S12. Scatter plots of observation values from PMF and prediction values from ML at three sites under (a)-(c) XGBoost; (d)-(f) LightGBM; (g)-(i) RF; (j)-(l) the time series of prediction and observation by XGBoost and PMF at three sites.

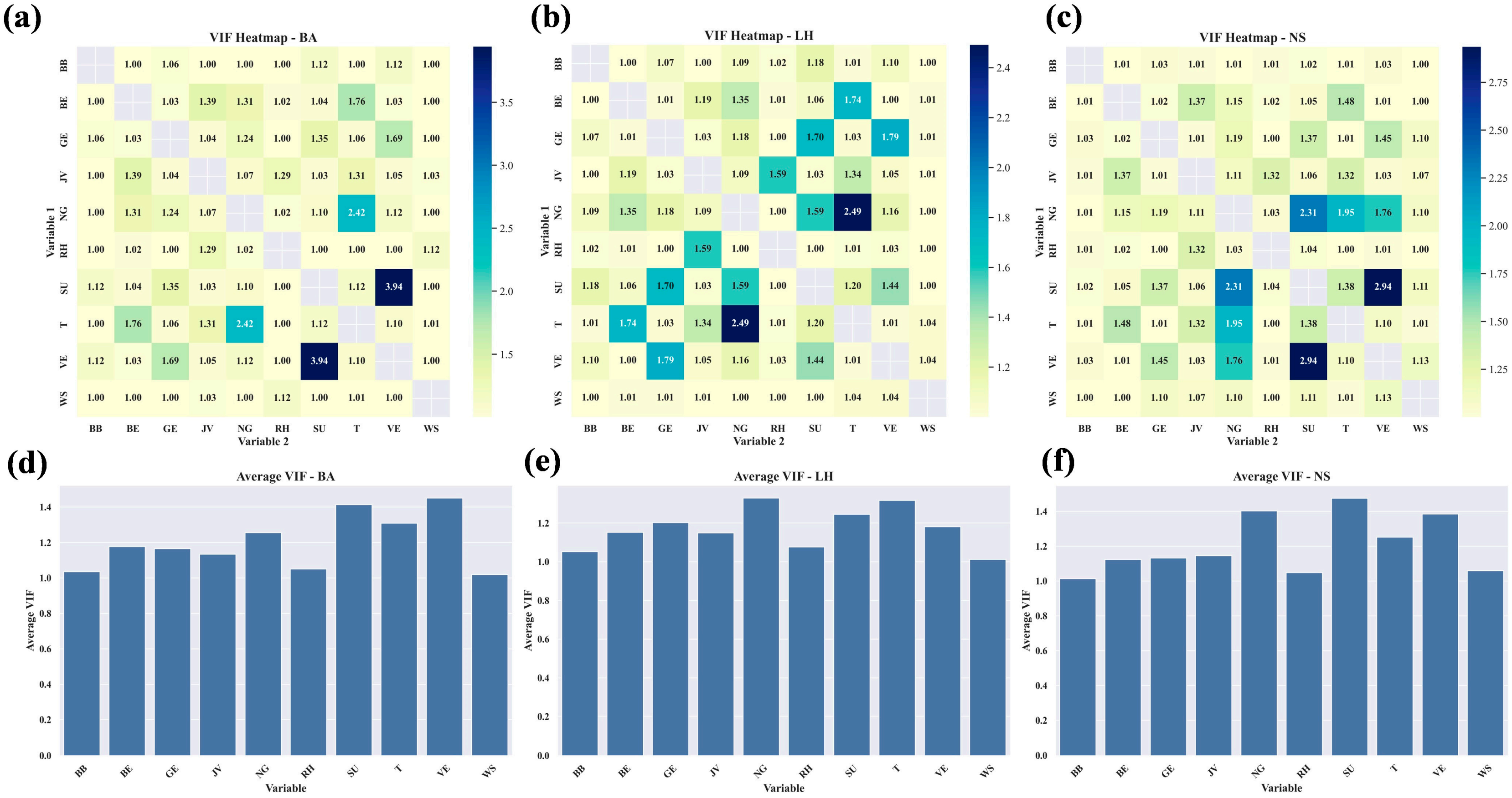

Figure S13. Heatmap and bar charts of variance inflation factor (VIF) between multiple factors output from the SHAP values.

Table S1. The information of the monitoring sites.

Site	Longitude	Latitude	Characteristic	Monitoring species
BA	113.764°E	22.729°N	Industrial site, industrial zones in the east and south, industrial cities in the north airport in the south, high-traffic expressways in the west and north, coast in the west	VOCs, Temperature (T), Relative humidity (RH), Wind speed (WS), Photolysis rate (J-value)
LH	113.994°E	22.703°N	Urban site (confounding region), industrial zones in the south and west, residential area in the southeast, reservoir and mountain in the east and north, adjacent traffic road in the south	VOCs, Temperature (T), Relative humidity (RH), Wind speed (WS), Photolysis rate (J-value)
NS	113.918°E	22.516°N	Urban site (city center), commercial areas in the southeast, urban park in the southwest, industrial zones in the northwest, residential areas in the east and north, traffic roads all around	VOCs, Temperature (T), Relative humidity (RH), Wind speed (WS), Photolysis rate (J-value)

Table S2. The concentration levels of monitoring species.

Species	Concentrations (ppb)		
	BA	LH	NS
**Alkanes (29)**	**12.987**	**12.956**	**9.692**
2,2-Dimethylbutane	0.054	0.045	0.031
2,3-Dimethylbutane	0.095	0.077	0.050
2-Methylpentane	0.432	0.396	0.260
3-Methylpentane	0.283	0.250	0.195
n-Hexane	0.418	0.372	0.245
2,4-Dimethylpentane	0.038	0.030	0.021
Methylcyclopentane	0.116	0.105	0.077
2-Methylhexane	0.223	0.178	0.076
2,3-Dimethylpentane	0.086	0.068	0.034
Cyclohexane	0.197	0.164	0.088
2,2,4-Trimethylpentane	0.029	0.017	0.028
n-Heptane	0.230	0.187	0.082
Methylcyclohexane	0.105	0.090	0.047
2,3,4-Trimethylpentane	0.020	0.009	0.020
2-Methylheptane	0.040	0.023	0.023
3-Methylheptane	0.044	0.023	0.022
n-Octane	0.047	0.032	0.034
Nonane	0.043	0.023	0.046
n-Decane	0.048	0.023	0.030
n-Undecane	0.018	0.023	0.026
n-Dodecane	0.011	0.006	0.016
Ethane	1.925	1.881	2.275
Propane	3.080	3.049	2.249
iso-Butane	1.498	1.802	1.060
n-Butane	2.412	2.789	1.533
Cyclopentane	0.048	0.034	0.043
iso-Pentane	0.740	0.679	0.659
n-Pentane	0.424	0.354	0.330
3-Methylhexane	0.282	0.228	0.090
**Alkenes (12)**	**2.699**	**2.244**	**2.371**
1,3-Butadiene	0.092	0.086	0.091
1-Pentene	0.020	0.018	0.024
trans-2-Pentene	0.027	0.012	0.032
Isoprene	0.490	0.444	0.435

cis-2-Pentene	0.036	0.017	0.028
trans-1,2-Dichloroethene	0.015	0.008	0.012
1-Hexene	0.021	0.017	0.032
Ethylene	1.065	0.949	1.000
Propylene	0.378	0.285	0.303
trans-2-butene	0.160	0.089	0.111
1-Butene	0.160	0.171	0.102
cis-2-Butene	0.236	0.148	0.201
**Aromatics (17)**	**5.823**	**4.408**	**3.970**
Benzene	0.291	0.255	0.229
Toluene	2.302	1.818	0.837
Ethylbenzene	0.357	0.227	0.261
m-Xylene	1.311	0.841	0.979
o-Xylene	0.460	0.324	0.359
Styrene	0.753	0.793	0.771
Isopropylbenzene	0.026	0.016	0.032
n-Propylbenzene	0.021	0.007	0.031
m-Ethyl toluene	0.044	0.021	0.080
p-Ethyl toluene	0.029	0.009	0.049
1,3,5-Trimethylbenzene	0.060	0.015	0.089
o-Ethyl toluene	0.024	0.010	0.042
1,2,4-Trimethylbenzene	0.075	0.038	0.105
1,2,3-Trimethylbenzene	0.028	0.015	0.041
m-Diethylbenzene	0.009	0.002	0.013
p-Diethylbenzene	0.013	0.007	0.019
Naphthalene	0.020	0.011	0.033
**Halohydrocarbons (34)**	**6.489**	**6.024**	**4.606**
Freon-12	0.440	0.459	0.498
Freon-114	0.017	0.018	0.021
Chloromethane	0.576	0.638	0.547
Vinylchloride	0.007	0.007	0.008
Bromomethane	0.013	0.011	0.014
Chloroethane	0.017	0.016	0.017
Freon-11	0.230	0.210	0.243
1,1-Dichloroethene	0.011	0.007	0.010
Freon-113	0.078	0.074	0.075
Dichloromethane	3.721	3.507	2.045
1,1-Dichloroethane	0.013	0.012	0.014
cis-1,2-Dichloroethene	0.007	0.006	0.007

Chloroform	0.191	0.201	0.225
1,1,1-Trichloroethane	0.007	0.002	0.008
CarbonTetrachloride	0.090	0.081	0.093
1,2-Dichloroethane	0.297	0.346	0.276
Trichloroethylene	0.357	0.200	0.076
1,2-Dichloropropane	0.106	0.101	0.088
Bromodichloromethane	0.010	0.002	0.013
trans-1,3-Dichloropropene	0.010	0.001	0.013
cis-1,3-Dichloropropene	0.009	0.001	0.013
1,1,2-Trichloroethane	0.017	0.011	0.026
Tetrachloroethylene	0.115	0.078	0.057
Chlorodibromomethane	0.012	0.001	0.017
1,2-Dibromoethane	0.010	0.001	0.016
Chlorobenzene	0.017	0.005	0.023
Bromoform	0.021	0.007	0.028
1,1,2,2-Tetrachloroethane	0.013	0.001	0.021
1,3-Dichlorobenzene	0.014	0.008	0.018
2-chlorotoluene	0.010	0.001	0.012
1,2-Dichlorobenzene	0.011	0.001	0.017
1,4-Dichlorobenzene	0.015	0.007	0.025
1,2,4-Trichlorobenzene	0.011	0.002	0.017
Hexachloro-1,3-butadiene	0.017	0.003	0.025
**OVOCs (21)**	**13.758**	**12.724**	**10.344**
Acetaldehyde	1.986	1.998	2.371
Acrolein	0.224	0.230	0.273
Propanal	0.324	0.311	0.333
Acetone	3.013	3.790	2.959
Isopropyl alcohol	0.869	1.100	0.443
MTBE	0.114	0.089	0.138
Methacrolein	0.104	0.104	0.098
Vinyl acetate	0.019	0.012	0.017
n-Butanal	0.250	0.201	0.252
MethylEthylKetone	1.247	0.950	0.762
Ethyl acetate	3.153	2.312	1.122
Tetrahydrofuran	0.064	0.046	0.038
Crotonaldehyde	0.067	0.052	0.067
Pentanal	0.116	0.119	0.152
Methyl methacrylate	0.056	0.034	0.030
1,4-Dioxane	0.029	0.005	0.029

Methyl isobutyl ketone	0.095	0.052	0.039
C6 ketones	0.025	0.006	0.021
Hexanal	1.875	1.159	1.050
Benzaldehyde	0.101	0.114	0.095
m-tolualdehyde	0.028	0.041	0.055
**Others (3)**	**1.221**	**1.209**	**1.182**
Acetylene	1.018	0.871	0.989
Carbon disulfide	0.072	0.266	0.054
Acetonitrile	0.131	0.072	0.138

Table S3. The list of representative species for PMF.

PMF species	*MDL* (ppb)			K_{OH}	*EF*
	BA	LH	NS	($\times10^{-12}$ cm^3 $molecule^{-1}$ s^{-1})	(%)
Ethane	0.041	0.072	0.034	0.25	10%
Propane	0.026	0.083	0.020	1.09	10%
i-Butane	0.018	0.082	0.018	2.12	10%
n-Butane	0.019	0.051	0.020	2.36	10%
i-Pentane	0.012	0.089	0.021	3.60	10%
n-Pentane	0.009	0.079	0.019	3.80	10%
2-Methylpentane	0.020	0.026	0.014	5.20	20%
3-Methylpentane	0.014	0.016	0.015	5.20	20%
n-Hexane	0.023	0.026	0.028	5.20	20%
Cyclohexane	0.018	0.016	0.023	6.97	20%
n-Heptane	0.020	0.018	0.018	6.76	20%
n-Octane	0.009	0.008	0.009	8.11	20%
Ethylene	0.048	0.096	0.039	8.52	20%
Propylene	0.069	0.175	0.051	26.30	20%
Acetylene	0.014	0.036	0.020	0.76	10%
Benzene	0.029	0.025	0.024	1.22	10%
Toluene	0.025	0.023	0.024	5.63	20%
Ethylbenzene	0.024	0.189	0.027	7.00	20%
m,p-Xylene	0.063	0.411	0.100	18.70	20%
o-Xylene	0.027	0.190	0.028	13.60	20%
Styrene	0.055	0.184	0.023	58.00	30%
MTBE	0.011	0.012	0.010	2.94	10%
Chloromethane	0.050	0.029	0.028	\	10%
Dichloromethane	0.023	0.013	0.017	\	20%
Acetonitrile	0.015	0.015	0.015	\	10%
Isoprene	0.020	0.021	0.019	100.00	\

Table S4. The test results of PMF.

Diagnostic	4	5	6	7	BA	LH	NS
Qrobust	607831	509027	422134	353551	562806	304451	509027
Qture	652441	540729	449301	376436	618419	324272	540729
Qr/Qt	0.93	0.94	0.94	0.94	0.91	0.94	0.94
Qexpeceted	166430.0	158475.0	150520.0	142565.0	163902.0	121405.0	158475.0
Q(true)/Qexp	3.92	3.41	2.98	2.64	3.77	2.67	3.41
DISP %dQ	<0.1%	<0.1%	<0.1%	<0.1%	<0.1%	<0.1%	<0.1%
DISP swaps	0	0	0	0	0	0	0
Number of BS run	100	100	100	100	100	100	100
Factors with the lowest BS mapping	98	94	53	74	94	89	94
BS-DISP% cases with swaps	0	0	0	0	0	0	0

Table S5. Ratio results of key species using characteristic ratio method.

Sites	i-Pentane/n-Pentane	i-Butane/n-Butane	Propane/Ethane	Toluene/Benzene
BA	2.45	0.69	1.67	6.83
LH	2.83	0.77	1.72	6.90
NS	2.26	0.73	0.95	3.27

Table S6. Machine learning outputs test results at various intervals for HCHO by XGBoost at three sites.

Interval for HCHO	BA				LH				NS			
(h)	R^2	*RMSE*	*MSE*	*MAE*	R^2	*RMSE*	*MSE*	*MAE*	R^2	*RMSE*	*MSE*	*MAE*
0.0	0.760	0.968	0.937	0.672	0.727	0.994	0.989	0.706	0.761	0.950	0.902	0.644
0.5	0.814	0.836	0.700	0.587	0.801	0.903	0.816	0.647	0.785	0.893	0.798	0.614
1.0	0.830	0.789	0.623	0.560	0.807	0.801	0.641	0.558	0.820	0.848	0.719	0.570
1.5	0.852	0.761	0.579	0.539	0.817	0.840	0.706	0.608	0.808	0.854	0.729	0.585
2.0	0.828	0.816	0.666	0.572	0.752	0.918	0.842	0.631	0.765	1.070	1.145	0.659
2.5	0.756	0.921	0.849	0.649	0.756	0.921	0.849	0.649	0.776	0.938	0.880	0.640
3.0	0.731	0.989	0.978	0.717	0.731	0.989	0.978	0.717	0.781	0.964	0.930	0.679

Table S7. Machine learning outputs test results under optimal conditions by various models at three sites.

Model	BA				LH				NS			
	R^2	*RMSE*	*MSE*	*MAE*	R^2	*RMSE*	*MSE*	*MAE*	R^2	*RMSE*	*MSE*	*MAE*
XGBoost	0.830	0.789	0.623	0.560	0.807	0.801	0.641	0.558	0.820	0.848	0.719	0.570
LightGBM	0.819	0.814	0.663	0.588	0.787	0.842	0.709	0.596	0.789	0.919	0.844	0.619
Random Forests(RF)	0.807	0.840	0.706	0.598	0.789	0.838	0.703	0.593	0.789	0.920	0.846	0.612

References

[1] He, Z.; Wang, X.; Ling, Z.; Zhao, J.; Guo, H.; Shao, M.; Wang, Z. Contributions of different anthropogenic volatile organic compound sources to ozone formation at a receptor site in the Pearl River Delta region and its policy implications. *Atmos. Chem. Phys.* **2019**, *19* (13), 8801–8816.

[2] Tong, M. X.; Zhang, Y. L.; Zhang, H. Y.; Chen, D. H.; Pei, C. L.; Guo, H.; Song, W.; Yang, X.; Wang, X. M. Contribution of Ship Emission to Volatile Organic Compounds Based on One-Year Monitoring at a Coastal Site in the Pearl River Delta Region. *J. Geophys. Res. Atmos.* **2024**, *129*, JD039999.

[3] Fang, H.; Huang, X. Q.; Xiao, S. X.; Lowther, S.; Fu, X. W.; Zhang, Y. L., Wu, T.; Hu, W. W.; Zhang, G. H.; Ding, X.; Tang, M. J.; Bi, X. H.; Jones, K. C.; Wang, X. M. Intermediate-volatility organic compounds observed in a coastal megacity: Importance of non-road source emissions. *J. Geophys. Res. Atmos.* **2022**, *127* (19), JD037301.

[4] McKeen, S.A.; Liu, S.C. Hydrocarbon ratios and photochemical history of. air masses. *Geophys. Res. Lett.* **1993**, *20* (21), 2363–2366.

[5] Atkinson, R.; Arey, J. Atmospheric degradation of volatile organic compounds. *Chem. Rev.* **2003**, *103* (12), 4605–4638.

[6] Atkinson, R.; Baulch, D. L.; Cox, R. A.; Crowley, J. N.; Hampson, R. F.; Hynes, R. G.; Jenkin, M. E.; Rossi, M. J.; Troe, J.; Subcommittee, I. Evaluated kinetic and photochemical data for atmospheric chemistry: Volume II – gas phase reactions of organic species. *Atmos. Chem. Phys.* **2006**, *6*, 3625-4055.

[7] Wu. Y. J.; Fan, X. L.; Liu, Y.; Zhang, J. Q.; Wang, H.; Sun, L.; Fang, T. G.; Mao, H. J.; Hu, J.; Wu, L.; Peng, J. F.; Wang, S. L. Source apportionment of VOCs based on photochemical loss in summer at a suburban site in Beijing. *Atmos. Environ.* **2023**, *293*, 119459.

[8] Parrish, D. D.; Stohl, A.; Forster, C.; Atlas, E. L.; Blake, D. R.; Goldan, P. D.; Kuster, W. C.; de Gouw, J. A. Effects of mixing on evolution of hydrocarbon ratios in the troposphere. *J. Geophys. Res. Atmos.* **2007**, *112*, JD007583.

[9] Zheng, H.; Kong, S. F.; Yan, Y. Y.; Chen, N.; Yao, L. Q.; Liu, X.; Wu, F. Q.; Cheng, Y.; Niu, Z. Z.; Zheng, S. R.; Zeng, X.; Yan, Q.; Wu, J.; Zheng, M. M; Liu, D. T.; Zhao, D. L.; Qi, S. H. Compositions, sources and health risks of ambient volatile organic compounds (VOCs) at a petrochemical industrial park along the Yangtze River. *Sci. Total Environ.* **2020**, *703*, 135505.

[10] Wang, Z. Y.; Shi, Z. B.; Wang, F.; Liang, W. Q.; Shi, G. L.; Wang, W. C.; Chen, D.; Liang, D. N.; Feng, Y. C.; Russell, A. G. Implications for ozone control by understanding the survivor bias in observed ozone-volatile organic compounds system. *Npj Clim. Atmos. Sci.* **2022**, *39*.

[11] Wei, D. D.; Cao, C.; Karambelas, A.; Mak, J.; Reinmann, A.; Commane, R. High-Resolution Modeling of Summertime Biogenic Isoprene Emissions in New York City. *Environ. Sci. Technol.* **2024**, *58*, 13783-13794.

[12] Stroud, C. A.; Roberts, J. M.; Goldan, P. D.; Kuster, W. C.; Murphy, P. C.; Williams, E. J.; Hereid, D.; Parrish, D.; Sueper, D.; Trainer, M.; Fehsenfeld, F. C.; Apel, E. C.; Riemer, D.; Wert, B.; Henry, B.; Fried, A.; Martinez-Harder, M.; Harder, H.; Brune, W. H.; Li, G.; Xie, H.; Young, V. L. Isoprene and its oxidation products, methacrolein and methylvinyl ketone, at an urban forested site during the 1999 Southern Oxidants Study. *J. Geophys. Res. Atmos.* **2001**, *106*, 8035–8046.

[13] Paatero, P.; Tapper, U. Positive matrix factorization: A non-negative factor model with optimal utilization of error estimates of data values. *Environmetrics* **1994**, *5* (2), 111–126.

[14] Paatero, P.; Hopke, P. K.; Song, X.; Ramadan, Z. Understanding and controlling rotations in factor analytic models. *Chemometr. Intel. Lab.* **2002**, *60*, 253–264.

[15] Peng, X.; Huang, X. F.; Wei, F. H.; Yan, R. H.; Tang, M. X.; Ji, J. P.; He, L.Y. Identifying the key drivers in retrieving blue sky during rapid urbanization in Shenzhen, China. *J. Clean. Prod.* **2022**, *356*, 131829.

[16] *EPA Positive Matrix Factorization (PMF) 5.0 Fundamentals and User Guide*; EPA/600/R-14/108; Office of Research and Development, U.S. Environmental Protection Agency: Washington, DC, 2014.

[17] Brown, S. G.; Eberly, S.; Paatero, P.; Norris, G. A. Methods for estimating uncertainty in PMF solutions: Examples with ambient air and water quality data and guidance on reporting PMF results. *Sci. Total Environ.* **2015**, *518–519*, 626–635.

[18] Zhu, B.; Cao, L. M.; Xia, S. Y.; Niu, Y. B.; Man, H. Y.; Du, K.; Yu, K. Y.; Huang, X. F. Identifying the airport as a key urban VOC source in the Pearl River Delta, China. *Atmos. Environ.* **2023**, *301*, 119721.

[19] Carter, W. P. L. Development of Ozone Reactivity Scales for Volatile Organic Compounds. *Air & Waste* **1994**, *44* (7), 881-899.

[20] Carter, W. P. L. Development of the SAPRC-07 chemical mechanism. *Atmos. Environ.* **2010**, *44* (40), 5324–5335.

[21] Chen, T. Q.; Guestrin, C. XGBoost: A Scalable Tree Boosting System. *arXiv* **2016**, *1603*, 02754.

[22] Ogata, S.; Takegami, M.; Ozaki, T.; Nakashima, T.; Onozuka, D.; Murata, S.; Nakaoku, Y.; Suzuki, K.; Hagihara, A.; Noguchi, T.; Iihara, K.; Kitazume, K.; Morioka, T.; Yamazaki, S.; Yoshida, T.; Yamagata, Y.; Nishimura, K. Heatstroke predictions by machine learning, weather information, and an all-population registry for 12- hour heatstroke alerts. *Nat. Commun.* **2021**, *12* (1), 4575.

[23] Chen, X.; Ma, W.; Zheng, F. X.; Wang, Z. C.; Hua, C. J.; Li, Y. R.; Wu, J.; Li, B. D.; Jiang, J. K.; Yan, C.; Petäjä, T.; Bianchi, F.; Kerminen, V. M.; Worsnop, D. R.; Liu, Y. C.; Xia, M.; Kulmala, M. Identifying Driving Factors of Atmospheric N_2O_5 with Machine Learning. *Environ. Sci. Technol.* **2024**, *58*, 11568-11577.

[24] Cheng, Y.; Huang, X. F.; Peng, Y.; Tang, M. X.; Zhu, B.; Xia, S. Y.; He, L. Y. A novel machine learning method for evaluating the impact of emission sources on ozone formation. *Environ. Pollut.* **2023**, *316*, 120685.

[25] Liu, X.; Lu, D. W.; Zhang, A. Q.; Liu, Q.; Jiang, G. B. Data-Driven Machine Learning in Environmental Pollution: Gains and Problems. *Environ. Sci. Technol.* **2022**, *56*, 2124-2133.

[26] Lundberg, S.; Lee, S. A. Unified approach to interpreting model predictions. *In Proceedings of the 31st International Conference on Neural Information Processing Systems*, **2017**, 4768-4777.

[27] Snoek, J.; Larochelle, H. Practical Bayesian Optimization of Machine Learning Algorithms. *arXiv* **2012**, *1206*, 2944.
